# Differential regulation of calcium-NFAT signaling pathway by Akt isoforms: unraveling effector dynamics and exhaustion of cytotoxic T lymphocytes in tumor microenvironment

**DOI:** 10.1136/jitc-2024-009827

**Published:** 2025-03-26

**Authors:** Wen-Ling Chen, Yong-Lin Chang, Su-Fang Lin, Ulrike Protzer, Masanori Isogawa, Hung-Chih Yang, Li-Rung Huang

**Affiliations:** 1 National Health Research Institutes, Institute of Molecular and Genomic Medicine, Miaoli, Taiwan; 2 National Health Research Institutes, National Institute of Cancer Research, Miaoli, Taiwan; 3 Institute of Virology, Technical University of Munich/ Helmholtz Munich, School of Medicine and Health, Munich, Germany; 4 Department of Virology II, National Institute of Infectious Diseases, Tokyo, Japan; 5 National Taiwan University Graduate Institute of Microbiology, Taipei City, Taiwan; 6 National Taiwan University Graduate Institute of Clinical Medicine, Taipei City, Taiwan; 7 Division of Gastroenterology and Hepatology, Department of Internal Medicine, National Taiwan University Hospital, Taipei, Taiwan

**Keywords:** Hepatocellular Carcinoma, T cell, Tumor microenvironment - TME, Adoptive cell therapy - ACT

## Abstract

**Background:**

Impairment of Akt signaling has been observed in antigen-specific cytotoxic T lymphocytes (CTLs) during chronic viral infections or tumor progression. Despite numerous studies emphasizing Akt’s role in driving CTL effector functions, there is limited exploration of using Akt molecules in T-cell engineering to enhance their antiviral or antitumor capabilities for therapeutic purposes. Some studies even conclude that inhibiting Akt activation during the in vitro expansion process can prevent T-cell exhaustion and boost the antitumor effector functions of chimeric antigen receptor-T cells in vivo. Given the unique expression patterns and functions of the three Akt isoforms in immune cells, we proposed that Akt isoforms in CTLs may regulate effector functions and T-cell exhaustion distinctly.

**Methods:**

In this study, we genetically modified tumor/virus-antigen-specific T-cell receptor tg CTLs to ectopically express Akt isoforms via retroviral transduction. We subsequently conducted western blotting, flow cytometry, and RNA sequencing analysis to assess their Akt expression, expression of immune checkpoints, antitumor/antivirus functionalities, and transcriptome. Additionally, we employed a persistent Hepatitis B Virus mouse model and a syngeneic hepatocellular carcinoma mouse model for further evaluation of their antivirus/antitumor efficacies.

**Results:**

We found that both Akt1 and Akt2 overexpression enhanced the cytotoxic capabilities of mouse CTLs, although with different dynamics. Specifically, Akt2 signaling in CTLs accelerated effector functions, leading to a rapid attack on tumor cells. Conversely, Akt1 signaling triggered calcium influx and subsequent nuclear factor of activated T cells (NFAT) activation, while Akt2 signaling suppressed calcium influx, preventing excessive NFAT expression and nuclear translocation. This repression of NFAT transcriptional activity by Akt2 signaling during prolonged antigen stimulation subsequently led to reduced expression of transcription factors associated with T-cell exhaustion, such as Egr2, Nr4a, Tox, and immune checkpoints. Consequently, Akt2-overexpressed CTLs displayed reduced T-cell exhaustion within the tumor microenvironment and efficiently eradicated tumors.

**Conclusion:**

These findings highlight the essential role of Akt signaling in enabling tumor-specific CTLs to eliminate cancer cells in the solid TME, with Akt isoforms differentially regulating the calcium–calcineurin–NFAT signaling pathway. This discovery suggests the potential of AKT2 in T-cell engineering technology to enhance the survival and effector functions of adoptively transferred T cells for treating liver malignancies or chronic viral infections.

**WHAT IS ALREADY KNOWN ON THIS TOPIC**

- Sustained Akt activation drives terminal effector CD8^+^ T-cell differentiation and limits central memory formation, but Akt signaling also supports differentiation of effector-like memory CD8^+^T cells. Although Akt1, Akt2, and Akt3 share 80% amino acid similarity, they have distinct functions in macrophage polarization, dendritic cell function, and T-cell differentiation. However, their specific roles in T-cell exhaustion remain unclear.

**WHAT THIS STUDY ADDS**

- This study demonstrates that Akt2, but not Akt1, prevents T-cell exhaustion by modulating calcium mobilization and nuclear factor of activated T cells activation in mouse cytotoxic T lymphocytes and a human T-cell line. We provide evidence that Akt2 suppresses prolonged calcium signaling, leading to reduced expression of exhaustion markers in cytotoxic T cells within the tumor microenvironment. These findings uncover a previously unrecognized mechanism by which Akt2 enhances antitumor immunity.

**HOW THIS STUDY MIGHT AFFECT RESEARCH, PRACTICE OR POLICY**

- Our findings suggest that targeting Akt2 signaling could improve T cell-based cancer immunotherapy by reducing T-cell exhaustion. This study provides a potential strategy to optimize chimeric antigen receptor-T cell and T-cell receptor-T cell therapies for solid tumors by fine-tuning calcium signaling. Future research could explore the therapeutic modulation of Akt2 to enhance T-cell persistence and functionality in cancer treatment.

## Background

 During T-cell activation, protein kinase B also known as Akt is phosphorylated at the threonine 308 and the serine 473 by phosphoinositide-dependent protein kinase 1 (PDK1) and mTOR Complex 2, respectively.^1^,^3^ Akt has a great influence on T-cell growth, proliferation, and survival and is a signal integrator for T-cell differentiation through Foxo, mTOR and Wnt/β-catenin pathways.^4^,^6^ Akt activation is indispensable for induction of effector functions of cytotoxic T lymphocytes (CTLs) during acute viral infection and is impaired in Ag-specific T cells during chronic viral infections or in malignancies.^7^ Sustained Akt activation tends to promote terminal differentiation of effector CD8^+^ T cells and impair the development of central memory T cells.^5^ However, a recent report indicated that Akt signaling facilitated the development and survival of CXCR3^lo^CD43^lo^ effector-like memory CD8^+^T cells.^8^ Virus or tumor-specific CD8^+^ T cells undergo T-cell exhaustion due to persistent T-cell receptor (TCR) signaling and lack of suitable co-stimulation^7 9 10^ in the context of chronic viral infection or malignancies, including Hepatitis B Virus (HBV)-specific T cells.^11 12^ T-cell exhaustion features the gradual loss of proliferative capability, impaired cytokine production, poor cytotoxicity, surface expression of various immune checkpoints and increase of apoptotic rate.^9 10 13^ Signaling through immune checkpoints on T cells could further change metabolic reprogramming during T-cell activation and differentiation.^14 15^ The ligation of programmed cell death protein 1 (PD-1) and cytotoxic T-lymphocyte-associated protein 4 (CTLA-4) on T cells is shown to suppress Akt activation on TCR stimulation.^16 17^

Although the three existing Akt isoforms (Akt1, Akt2, Akt3) share 80% similarity in amino acid structure, they show non-redundant physiological properties and also exhibit distinctive functions in several immune cells.^18^ Akt1 and Akt2 differentially regulate macrophage differentiation toward M2 and M1 phenotypes, respectively,^19^ as well as the development and function of dendritic cells.^20^ There are also reports showing that Akt isoforms differentially regulate Th1/Th17 responses during experimental autoimmune encephalomyelitis disease progression and Th1‐regulatory T cell (Treg) generation in autoimmune diseases.^21 22^ Pharmacological inhibition of Akt1 and Akt2 has been shown to enhance the central memory phenotype of CD8^+^ T cells while preventing terminal differentiation.^23^ However, their Akt1 and Akt2 knockout studies suggested that Akt1 and Akt2 differentially influence T-cell differentiation.^23^ These findings led us to hypothesize that Akt isoforms differentially regulate CTLs during chronic viral infection and tumor progression and that ectopic Akt expression may enhance their antiviral or antitumor effector functions.

In this study, we initially investigated the cell expansion, antiviral or antitumor functionalities and differentiation of intrahepatic and intratumoral antigen (Ag)-specific CTLs in HBV-infected liver or tumor microenvironment (TME) of hepatocellular carcinoma (HCC). Our results clearly demonstrated that ectopic expression of Akt1 or Akt2 in virus-specific CTLs enhanced their antiviral efficacy with strong cell expansion and cytotoxicity in response to intrahepatic viral Ag stimulation and clear viral infection. Although both Akt1 or Akt2 signaling prevented the expression of immune checkpoints, especially lymphocyte-activation gene 3 (LAG-3) and T-cell immunoreceptor with Ig and ITIM domains (TIGIT) on CTLs, only Akt2-OE CTLs but not Akt1-OE CTLs were able to eliminate tumor cells in the setting of the HCC mouse model. These Akt2-OE CTLs showed a greater capability to proliferate, to release cytokines and to kill tumor cells in comparison with Akt1-OE or control CTLs. Martin *et al* previously reported that Akt2 activation regulated the duration of calcium mobilization by reducing the time of calcium release from the endoplasmic reticulum without affecting the initial calcium amplitude, thereby inhibiting nuclear factor of activated T cells (NFAT) activation in a chicken B-lineage lymphoma cell line and in Jurkat T-cell line.^24^ Similarly, our study found that Akt2 signaling suppressed calcium influx and subsequently inhibited NFAT activation in CTLs on TCR stimulation. Furthermore, we demonstrated that ectopic expression of Akt2 prevented T-cell exhaustion caused by hyperactivation of NFAT signaling. These findings highlight that Akt2 signaling is indispensable for tumor-specific CTLs to effectively eliminate cancer cells in the solid TME, and also reveal that Akt isoforms differentially regulate the calcium–calcineurin–NFAT signaling pathway. This underscores the potential of Akt2 in T-cell engineering to enhance the survival and effector functions of adoptively transferred T cells, offering promising therapeutic strategies for liver malignancies and chronic viral infections.

## Methods

### Animal studies

Male C57BL/6J mice aged 5–6 weeks were purchased from the National Laboratory Animal Center in Taipei, Taiwan, and housed at the NHRI laboratory animal center, accredited by AAALAC International. HBc_93-100_-specific TCR transgenic (HBc TCR tg) mice (B6.Cg-*Ptprc^a^ Pepc^b^* Tg (TcraBC10,TcrbBC10)3Chi/J) were generously provided by Dr Francis V Chisari from The Scripps Institute, La Jolla, USA.^25^ The purchased mice were used 1 week after arrival at the NHRI Laboratory Animal Center.

For the establishment of a persistent HBV mouse model, C57BL/6J mice were intravenously injected with 1–3×10^8^ infectious units of recombinant adenovirus carrying the HBV genome (AdHBV) to induce intrahepatic persistent viral infections.^26^ HBV carrier mice with hepatitis B e antigen (HBeAg) titer above 100 S/CO were grouped evenly and served as recipients for adoptive T-cell transfer 1-month post-infection. The details of the induction of the HCC mouse model were described previously^27^ and in online supplemental methods. Photon emission from transduced hepatocytes or tumor cells was monitored periodically using an In Vivo Imaging System (IVIS) imaging system (Caliper Life Sciences, Massachusetts, USA). HCC-bearing mice with a total photon flux from IVIS imaging exceeding 1×10^8^ photons/s were grouped evenly and used as recipients for adoptive T-cell transfer, while those with a total flux exceeding 3×10^10^ photons/s or experiencing a ≥20% weight loss were humanely euthanized to prevent suffering from large liver tumors.

### Plasmid constructs for viral production

Plasmids used to produce recombinant retroviruses carrying mouse *Akt1*, *Akt2*, *Akt3* or control *Cd90.1*, respectively, were generated through PCR cloning linking myristoylation sequence, mouse Akt1, Akt2 or Akt3, p2A peptide sequence, mouse CD90.1 and woodchuck hepatitis virus post-transcriptional regulatory element in mouse stem cell virus vector. CA-RIT-NFAT1 (Addgene #85181), DBDmut-CA-RIT-NFAT1 (Addgene #63669) and the mouse stem cell virus vector (MSCV) plasmids carrying Akt1-CD90.1, Akt2-CD90.1, Akt3-CD90.1 and control CD90.1 open reading frames, respectively, were transfected to platinum-E retroviral packaging cell line (Cell Biolabs, San Diego, USA) to produce ecotropic retroviruses for the transduction of mouse CTLs.

The MSCV plasmids carrying Akt1-CD90.1, Akt2-CD90.1 and control CD90.1 open reading frames, respectively, together with pMD.G (The RNAi Core, Taipei, Taiwan) were transfected to the 293-GP retroviral packaging cell line to produce VSV-G-pseudotyped retroviruses for the transduction of the Jurkat cell line.

A 7-copy NFAT binding DNA sequence (GGAGGAAAAACTGTTTCA), DNA sequences of minimal cytomegalovirus promoter and aequorea coerulescens green fluorescent protein (AcGFP1) open reading frame were synthesized and subcloned into lentiviral pLVX vector to result in pLVX-NFAT BS-miniP-AcGFP plasmid. The resultant plasmid together with pCMV-ΔR8.91 (The RNAi Core, Taipei, Taiwan) and pMD.G plasmids were transfected into 293T packaging cells to produce VSV-G-pseudotyped lentivirus for the establishment of NFAT reporter Jurkat cell line following the protocol provided by the RNAi core.

### T-cell preparation, retroviral transduction and adoptive transfer

All primary CD8^+^ T cells used in this study were derived from HBc TCR tg mice. Splenic CD8^+^ T cells from CD45.1^+^ HBc TCR tg mice were isolated via immunomagnetic separation using CD8 microbeads (STEMCELL Technologies, Vancouver, Canada). Purified CD8^+^ T cells were activated with anti-CD3/CD28-labeled T-activator Dynabeads (Thermo Fisher Scientific, Waltham, USA) in RPMI 1640 medium supplemented with 8% FCS, 50 µM 2-mercaptoethanol, glutamine, and antibiotics. 1-day post-stimulation, the activated CD8^+^ T cells were transduced with recombinant MSCV retroviruses as per the manufacturer’s instructions (Clontech Laboratories, Mountain View, California, USA). The transduced CTLs were used for adoptive transfer or underwent in vitro re-stimulation at day 3 post-primary stimulation for RNA isolation or surface marker analysis. Following magnetic removal of Dynabeads, live CTLs were separated from dead cells using Ficoll-Paque Plus density gradient media (GE HealthCare Life Sciences, Illinois, USA), washed twice in Dulbecco’s Phosphate Buffered Saline (DPBS) and resuspended in DPBS for intravenous injection into HBV carrier mice or HCC-bearing mice.

### Cell isolation and flow cytometric analysis

Spleens, draining lymph nodes, and liver/tumors were harvested from virus-infected or HCC-bearing mice at specified time points. The detailed procedures were described in online supplemental methods. All antibodies used for flow cytometry were obtained from commercial sources, and the specific clones, fluorochromes, and vendors are listed in online supplemental table S1.

### Detection of HBV antigens, anti-HBs, and serum alanine aminotransferase

Serum levels of HBeAg were determined using the Elecsys HBe Ag system (Roche Diagnostics, Mannheim, Germany). Serum alanine aminotransferase (ALT) activity was measured using specific bioreaction strips on a Reflovet Plus reader (Roche Diagnostics).

### In vivo T-cell proliferation assays

HCC-bearing mice receiving T-cell adoptive transfer were intravenously administered 1 mg of 5-ethynyl-2-deoxyuridine (EdU) at indicated time points post-adoptive transfer. 1 day after EdU injection, tumor-infiltrating leukocytes, splenocytes, and lymph node cells were harvested and subjected to surface marker staining. Additionally, EdU incorporation was detected using the Click-iT EdU Alexa Fluor 488 Flow Cytometry Assay Kit (Thermo Fisher Scientific) followed by flow cytometric analysis.

### In vitro T-cell assays

Genetically modified HBc_93-100_-specific CTLs were harvested at day 3 post-activation and used directly in Western blot and flow cytometric analysis or re-stimulated by anti-CD3/anti-CD28 beads for 6~24 hours and then used in FACS analysis for analyzing the expression of surface molecules or western blot. For detection of perforin and granzyme B in the adoptively transferred CTLs, the tumor-infiltrating leukocytes (TILs) were harvested from tumor tissue of HCC-bearing mice at indicated time points post-adoptive transfer and subjected directly to surface marker staining followed by intracellular staining of perforin and granzyme B. For flow cytometric analysis of the percentage of cytokine-secreting CTLs in tumors, the TILs were re-stimulated with HBc_93-100_ peptides (MGLKFRQL) for 6 hours in the presence of Brefeldin A (5 µg/mL; BioLegend) and Monensin (2 µM; BioLegend) for 5 hours and subjected to surface marker staining followed by intracellular staining of interferon (IFN)-γ and tumor necrosis factor (TNF)-α. CD8^+^CD45.1^+^ cells were gated and defined as transferred CTLs.

### Calcium mobilization assay

The genetically modified HBc_93-100_-specific CTLs, at day 4 post-activation, were initially labeled with 520 AM dye (Abcam, Cambridge, UK) in the assay buffer provided in the kit at 37°C for 30 min, spun down to remove the dye, and resuspended in 0.4 mL Hank's Buffer with Hepes (also provided in the kit) for further αCD3 or ionomycin stimulation and flow cytometric analysis to detect intracellular calcium mobilization (as illustrated in online supplemental figure S1).

### NFAT reporter assay

The Jurkat cell line was transduced with VSV-G-pseudotyped lentivirus carrying NFAT BS-miniP-AcGFP gene sequence. 1 week after transduction, transduced single cells were cultured individually and expanded into clones. A reporter clone was selected based on the low GFP background and high responsiveness to turn into GFP-positive toward TCR triggering. The selected NFAT reporter cells were transduced with VSV-G-pseudotyped retroviruses carrying *Akt1*, *Akt2* or control *Cd90.1*, respectively. The transduction efficiency of each group was greater than 95%. 5 days post transduction, until the transgenes were stably expressed by the reporter cells, the reporter cells were restimulated with plate-bound anti-CD3 (Ab clone: OKT3; at indicated concentration) and anti-CD28 (Ab clone: CD28.2; 1 mg/mL for coating) for the indicated time period. The percentage or the absolute cell numbers of GFP^+^ cells were analyzed by flow cytometry or by IncuCyte S3 live-cell analysis system (Sartorius, Germany).

### Immunohistochemistry study

Paraffin-embedded liver/tumor tissue sections were deparaffinized, rehydrated, followed by heat-induced antigen retrieval and then incubated with primary antibodies as listed in online supplemental table S1. The signals were visualized using horseradish peroxidase-conjugated secondary Abs and 3,3′-Diaminobenzidine (DAB) peroxidase substrate kit as described in the online supplemental methods.

### RNA sequencing and bioinformatics

RNA sequencing (RNA-seq) was performed on 18 RNA samples belonging to six groups (ctrl, Akt1-OE, Akt2-OE, Re-ctrl, Re-Akt1-OE, and Re-Akt2-OE) with three samples in each group. Transcriptome library construction and sequencing were conducted on RNAs from each group (Biotools, New Taipei City, Taiwan). The Illumina sequencer generated raw data that was transformed into sequenced reads using CASAVA software (V.1.8), followed by the removal of bad reads and adaptors. Clean reads were mapped to the murine genome (mm10) using TopHat2 (V.2.0.12). HTSeq software (V.0.6.1, union mode) was used to quantify gene expression levels and represent them as fragments per kilobase of transcript per million mapped reads. The R package limma (V.3.52.2) was employed to identify differentially expressed genes from the normalized expression matrices, and visualization was achieved using R pheatmap (V.1.0.12). To identify biological pathways enriched in the Akt1-OE and Akt2-OE CTLs, Gene Set Enrichment Analysis (GSEA)^28 29^ was performed on the expression matrix of the restimulated group (n=9) against a custom gmt matrix comprising 86 selected gene sets of the C7 immunologic signature database along with Mouse_Gene_Symbol_Remapping_Human_Orthologs_MSigDB.v2024.1.Hs.chip as chip platform.

### Statistical analysis

All statistical analyses were performed using GraphPad Prism V.7 or V.10 (GraphPad Software, La Jolla, USA). Data are presented as mean±SD unless otherwise specified. For comparisons between two groups, an unpaired two-tailed Student’s t-test was used. For longitudinal tumor growth comparisons, a mixed-effects model analysis was applied to account for repeated measurements over time. The specific statistical tests and sample sizes (n) for each experiment are detailed in the corresponding figure legends.

## Result

### Akt1 and Akt2 isoform signaling in CTLs orchestrate similar antiviral but divergent antitumor responses in the liver

**Figure 1:**
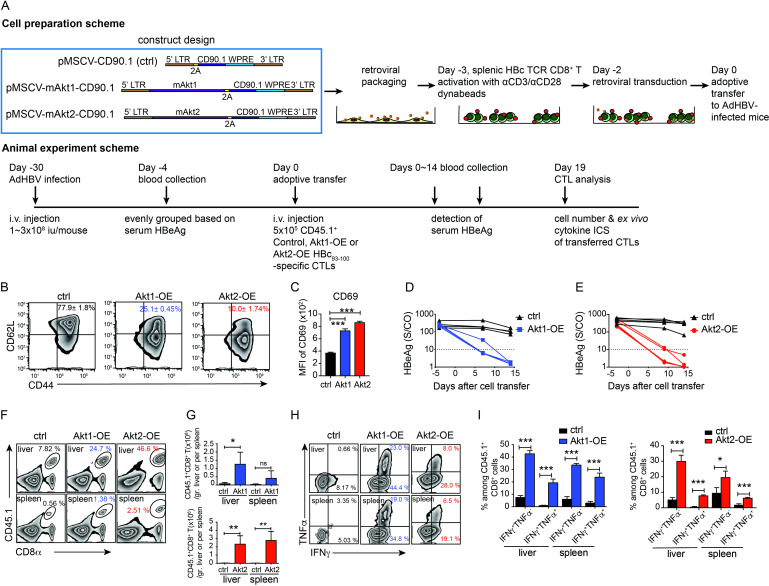
Akt signaling in CTLs promoted antiviral responses against HBV in a persistent HBV mouse model. (A) Schematic representation of the MSCV retroviral constructs encoding Akt1, Akt2, and Cd90.1 reporter genes used for T-cell engineering. The timeline of experimental procedures is illustrated. (B) Representative flow cytometry plots show the expression of CD62L and CD44 in control, Akt1-OE, and Akt2-OE CTLs before adoptive transfer. Percentages indicate the proportion of CD44^+^CD62L^+^ cells in each group. (C) The mean fluorescence intensity (MFI) of CD69 in control, Akt1-OE, and Akt2-OE CTLs before adoptive transfer detected by flow cytometry. (D) Kinetics of serum HBeAg levels of HBV carrier mice receiving the adoptive transfer of 5×10^5^ control- or Akt1-OE CTLs. (n=4 per group) (E) Kinetics of serum HBeAg levels of HBV carrier mice receiving the adoptive transfer of 5×10^5^ control or Akt2-OE CTLs. (n=5 per group) (F) Gating and (G) cell numbers of CD45.1^+^ transferred CTLs in livers and spleens of HBV carrier mice as in D and E at day 19 post adoptive transfer. (H, I) Cytokine production of control, Akt1-OE, or Akt2-OE CTLs isolated from the liver and spleen of HBV carrier mice on day 19 post-transfer. CTLs were stimulated ex vivo with HBc_93-100_ peptide for 6 hours, and intracellular IFN-γ and TNF-α levels were analyzed by flow cytometry. Zebra plots show representative data, and bar graphs display the percentages of IFN-γ^+^ CTLs or double-positive (IFN-γ^+^TNF-α^+^) CTLs among CD45.1^+^CD8^+^ T cells. Statistical analysis was performed using unpaired Student’s t-test, and significance is indicated as *p<0.05, **p<0.01, and ***p<0.001. AdHBV, adenovirus carrying the HBV genome; HBV, hepatitis B virus; CTLs, cytotoxic T lymphocytes; HBeAg, hepatitis B e antigen; ICS, intracellular staining; IFN, interferon; i.v., intravenous; pMSCV, post in mouse stem cell virus vector; TNF, tumor necrosis factor.

Akt activation is often impaired in antigen-specific T cells during chronic viral infections or in tumor antigen-specific T cells in the TME.^16^ This observation prompted us to explore the impact of Akt signaling on the antiviral or antitumor functionalities of CTLs. We generated four constructs using PCR cloning and produce retroviruses for genetic modification of mouse CD8^+^ T cells (figure 1A construct design and online supplemental figure S2A). Following retroviral transduction (figure 1A cell preparation scheme), a high proportion (75–95%) of CD8^+^ T cells transduced with Cd90.1, Akt1-Cd90.1, or Akt2-Cd90.1 expressed CD90.1 as confirmed by surface staining and flow cytometry. However, only 23% of CD8^+^ T cells transduced with Akt3-Cd90.1 expressed CD90.1 at low levels (online supplemental figure S2B), likely reflecting the tissue-specific expression of Akt3 in the brain and testes.^30^ Consistent with previous studies, both mouse primary T cells and human Jurkat T cells predominantly express Akt1 and Akt2, with little to no expression of Akt3.^31^ Western blot analysis confirmed the presence of exogenous myristoylated Akt isoforms in Akt-OE CTLs, which were absent in control T cells. Phosphorylation at Ser473 was observed in all Akt isoform-OE CTLs, whereas phosphorylation at Thr308 occurred only in Akt1-OE or Akt2-OE (online supplemental figures S2C and S3).

The Akt1-OE or Akt2-OE CTLs specific to HBV core Ag 93–100 peptide (HBc_93-100_) were analyzed for memory and activation marker expression before adoptive transfer into AdHBV-infected mice^26^ and analyzed their antiviral functionalities after adoptive transfer (figure 1A animal experiment scheme). Akt1-OE and Akt2-OE CTLs before adoptive transfer showed reduced CD62L levels but elevated CD69 expression compared with control CTLs (figure 1B,C). Both Akt1-OE and Akt2-OE CTLs but not control CTLs completely cleared persistent HBV infection within 14 days (figure 1D,E) and exhibited robust cell expansion in the liver and spleen after transfer (figure 1F,G). Leukocytes from the liver and spleen were re-stimulated with HBc_93-100_ peptides 19 days after transfer. Akt1-OE and Akt2-OE CTLs exhibited significantly enhanced IFN-γ and TNF-α production compared with control CTLs (figure 1H,I). Building on previous findings that Akt signaling promotes CD8^+^ T-cell differentiation into effector cells,^5 23^ our study further demonstrates that Akt overexpression effectively consolidates antiviral T-cell immunity within the immunosuppressive liver microenvironment.

**Figure 2:**
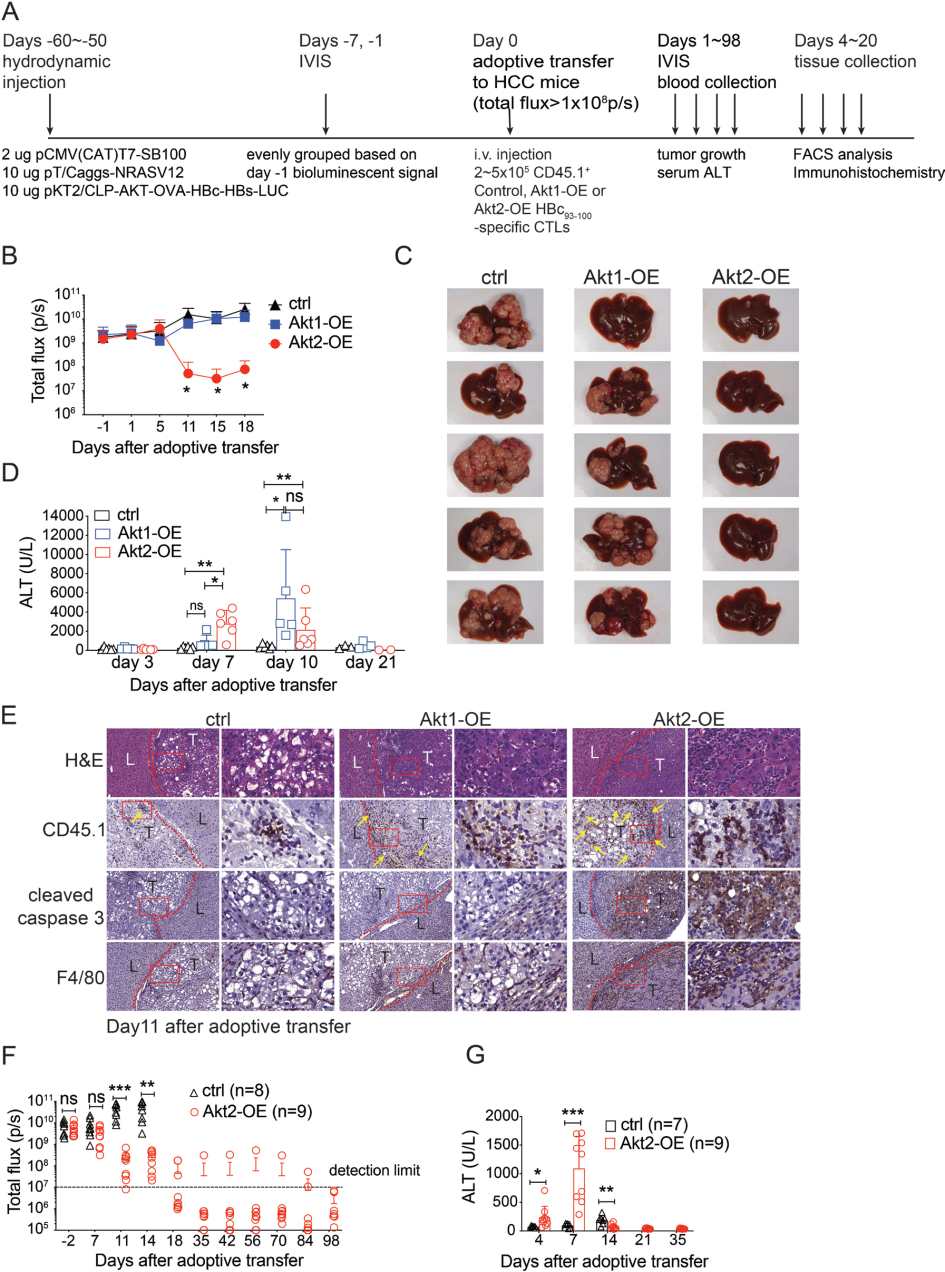
Akt2-OE tumor-specific CTLs exhibited superior anti-HCC capability. (A) Schematic representation of the animal experimental workflow for generating HCC-bearing mice and adoptive cell transfer. (B) In vivo bioluminescence of HCC-bearing mice receiving 2×10^5^ control, Akt1-OE or Akt2-OE CTLs. (n=5 or 6 per group). (C) Liver images of HCC-bearing mice receiving 2×10^5^ control, Akt1-OE or Akt2-OE CTLs at day 19 post-adoptive transfer. (D) Kinetics of serum ALT in mice receiving 2×10^5^ control, Akt1-OE or Akt2-OE CTLs. (E) H&E staining and immunohistochemical staining of the tumor/liver sections for the congenic marker of adoptively transferred CTLs (CD45.1), cleaved caspase 3 and F4/80. (F) In vivo bioluminescence of long-term follow-up of HCC-bearing mice receiving 2×10^5^ control or Akt2-OE CD8^+^ T cells. (G) Serum ALT levels of long-term follow-up of HCC-bearing mice as in F. Statistical analysis was performed using unpaired Student’s t-test, and significance is indicated as *p<0.05, **p<0.01, and ***p<0.001. ALT, alanine aminotransferase; CTLs, cytotoxic T lymphocytes; HCC, hepatocellular carcinoma; i.v., intravenous; IVIS, In Vivo Imaging System.

We then investigated whether Akt1 or Akt2 signaling also enabled the tumor-specific CTLs to overcome TME in the oncogene-induced HCC mouse model. This model was established through the hydrodynamic injection of plasmids carrying genes encoding transposase, human Akt1, N-Ras, surrogate tumor antigens (HBc_93-100_) and luciferase.^27^ The progression of liver cancers in mice before (day −1) and after T-cell transfer could be monitored using the IVIS (figure 2A). We observed a reduction in tumor size in the group of mice receiving Akt2-OE CTLs, but no significant change was seen in the groups receiving control or Akt1-OE CTLs (figure 2B,C and online supplemental figure S4). Mice receiving Akt2-OE CTLs exhibited higher serum ALT levels on day 7 compared with mice receiving control or Akt1-OE CTLs. However, some mice receiving Akt1-OE CTLs displayed extremely elevated ALT levels on day 10, despite no decrease in tumor burden (figure 2D). Histological examination of liver/tumor tissues revealed extensive infiltration of mononuclear cells in tumors of mice receiving Akt2-OE CTLs, while tumors of mice receiving control or Akt1-OE CTLs showed fewer mononuclear cells (figure 2E, H&E). Immunohistochemical staining confirmed the presence of CD45.1^+^ transferred T cells, showing that Akt2-OE CTLs could penetrate the tumor core, while control or Akt1-OE CTLs were primarily confined to the stroma (figure 2E, CD45.1 staining in brown, indicated by yellow arrow). Tumors of mice receiving Akt2-OE CTLs exhibited both tumor cell apoptosis and substantial macrophage infiltration (figure 2E, cleaved caspase 3 and F4/80 staining in brown), in contrast to the other groups. These findings suggest that Akt2 signaling induces prompt cytotoxicity in CTLs, allowing them to penetrate the tumor core, while Akt1 signaling in CTLs enhances delayed cytotoxicity in CTLs that remain in the stroma and the liver, leading to non-specific liver damage.

Most mice receiving Akt2-OE CTLs did not experience HCC recurrence within 98 days (figure 2F, online supplemental figure S5). Importantly, Akt2-OE CTLs only induced an acute antitumor response without causing chronic liver damage, as evidenced by the transient serum ALT elevation in HCC mice receiving Akt2-OE CTLs (figure 2D,G). Akt1-OE and Akt2-OE CTLs display distinct antitumor functionalities, indicating that Akt1 and Akt2 signaling confer varying degrees of resistance to the immunosuppressive TME. Therefore, it is worth investigating whether Akt isoforms differentially regulate T-cell fitness in the HCC TME and its underlying mechanism.

### Akt2 signaling enhances the Ag-specific expansion of CTLs in the HCC microenvironment

**Figure 3:**
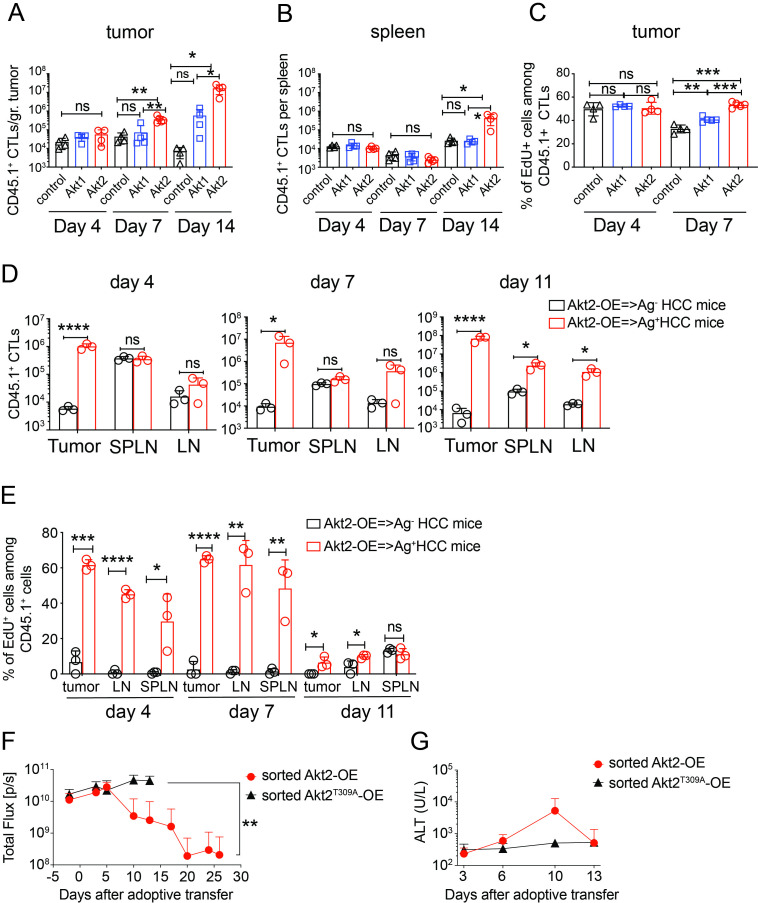
Akt2-CTLs proliferated and expanded vigorously in response to tumor Ag stimulation in the tumor microenvironment. (A) Time kinetics of cell number of adoptively transferred CD45.1^+^ CTLs in the tumor (A) or in the spleen (B) of HCC-bearing mice receiving 2×10^5^ control, Akt1-OE or Akt2-OE CTLs at days 4, 7 and 14 post-adoptive transfer. (C) Percentage of EdU-incorporated CD45.1^+^CTLs in the tumor of mice as in A. 1 mg of EdU was given intravenously at day 3 or day 6 post-adoptive transfer. The leukocytes were purified from the tumor masses of the mice harvested at day 4 or day 7 post the adoptive transfer and subjected to surface marker staining and EdU detection followed by flow cytometric analysis. (D) Adoptively transferred CD45.1^+^ Akt2-OE CTLs (5×10⁵) were infused into HBc_93-100_-expressing (Ag^+^) or HBc_93-100_-negative (Ag^−^) HCC-bearing mice. The numbers of CD45.1^+^ CTLs in tumors, spleens (SPLN), and draining lymph nodes (LN) were quantified on days 4, 7, and 11 post-transfer. (E) Proliferation of adoptively transferred CD45.1^+^ CTLs in tumors, SPLN, and draining LN was assessed on days 4, 7, and 11 post-transfer. EdU was injected intravenously on days 3, 6, and 10, and EdU incorporation was analyzed by flow cytometry. (F) Tumor growth was monitored in HCC-bearing mice receiving adoptive transfer of FACSorted 3.3×10^5^ Akt2-OE CTLs (n=9) or Akt2^T309A^-OE CTLs (n=5). Total tumor flux (photons/second) was measured by In Vivo Imaging System over 25 days post-transfer. (G) Serum alanine aminotransferase levels in HCC-bearing mice as described in F, measured at days 3, 6, 10, and 13 post-transfer. Statistical analysis was performed using unpaired Student’s t-test (A–E) or two-way analysis of variance (F), and significance is indicated as *p<0.05, **p<0.01, ***p<0.001, ****p<0.0001. Ag, antigen; CTLs, cytotoxic T lymphocytes; EdU, 5-ethynyl-2-deoxyuridine; HCC, hepatocellular carcinoma.

Leukocytes from tumors and spleens of HCC-bearing mice receiving control, Akt1-OE, or Akt2-OE CTLs were analyzed on day 4, day 7, and day 14 post-adoptive transfer. Intratumoral and splenic transferred T-cell numbers were similar across all groups on day 4. However, Akt2-OE CTLs expanded significantly in the tumor by day 7, surpassing control and Akt1-OE CTLs and this trend persisted through day 14 (figure 3A, online supplemental figure S6). Splenic Akt2-OE CTLs also showed significant expansion by day 14 compared with control or Akt1-OE CTLs (figure 3B, online supplemental figure S6). While proliferative capacity did not differ among intratumoral CTLs on day 4, proliferation declined in control and Akt1-OE CTLs by day 7, correlating with the sustained expansion of Akt2-OE CTLs (figure 3C, online supplemental figure S6). Despite moderate proliferative capabilities, Akt1-OE CTLs failed to expand or eliminate tumors, suggesting that Akt2 signaling promotes T-cell expansion and survival through mechanisms beyond proliferation alone.

To explore the role of antigen stimulation, Akt2-OE CTLs were transferred into mice with either antigen-positive (Ag^+^) or antigen-negative (Ag^−^) HCC. In Ag- HCC mice, Akt2-OE CTLs showed minimal proliferation and expansion in tumor, spleen, or lymph nodes across all time points (figure 3D,E, online supplemental figure S7). In contrast, in Ag^+^ HCC mice, Akt2-OE CTLs displayed robust proliferation as evidenced by EdU incorporation on days 4 and 7 and significant expansion in tumor tissues from day 4 to day 11 (figure 3D,E, online supplemental figure S7). These findings demonstrate that Akt2 signaling alone is insufficient to drive CTL proliferation without antigen-dependent TCR stimulation, emphasizing the safety of Akt2-modified T cells in immunotherapy.

Further, we investigated the functional importance of Akt2 kinase activity by introducing a threonine for alanine substitution at amino acid 309 of Akt2, which abolishes kinase activity.^32^ Sorted Akt2^T309A^-OE CTLs exhibited impaired antitumor activity, as evidenced by sustained bioluminescence signals and a lack of tumor regression, in contrast to the robust tumor clearance observed with Akt2-OE CTLs (figure 3F, online supplemental figure S7). Moreover, ALT levels in mice receiving Akt2^T309A^-OE CTLs were markedly lower than in mice receiving Akt2-OE CTLs, further supporting the functional importance of Akt2’s kinase activity (figure 3G). These findings confirm that Akt2 signaling promotes CTL expansion and antitumor activity through its kinase-dependent mechanisms, highlighting the critical role of Akt2 kinase activity in enhancing the efficacy of CTL-based immunotherapies.

### Akt2 signaling rapidly induces effector functions of CTLs in the TME

**Figure 4:**
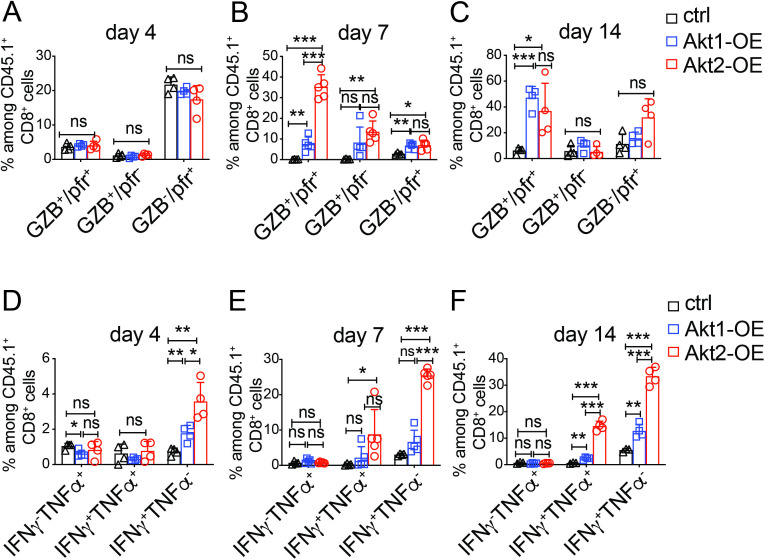
Akt2 signaling rapidly enhanced effector functions of CTLs in the tumor microenvironment. (A~C) Percentage of perforin and granzyme B expressing cells among CD45.1^+^ CTLs isolated from the tumor masses of HCC-bearing mice receiving 2×10^5^ control, Akt1-OE or Akt2-OE CTLs at day 4 (A), day 7 (B) and day 14 (C) post-adoptive transfer. (D~F) Percentage of cytokine-producing cells among CD45.1^+^ CTLs isolated from the tumor masses of HCC-bearing mice receiving 2×10^5^ ctrl, Akt1-OE or Akt2-OE CTLs at day 4 (D), day 7 (E) and day 14 (F) post-adoptive transfer. CTLs were stimulated ex vivo with HBc_93-100_ peptide for 6 hours, and intracellular IFN-γ and TNF-α levels were analyzed by flow cytometry. Bar graphs display the percentages of IFN-γ^+^, TNF-α^+^ or double-positive (IFN-γ^+^TNF-α^+^) CTLs among CD45.1^+^CD8^+^ T cells. Statistical analysis was performed using unpaired Student’s t-test, and significance is indicated as *p<0.05, **p<0.01, and ***p<0.001. CTLs, cytotoxic T lymphocytes; HCC, hepatocellular carcinoma; IFN, interferon; TNF, tumor necrosis factor.

Approximately 20% of the adoptively transferred CTLs in the TME expressed perforin, and less than 5% expressed granzyme B at day 4 after infusion, with few cells co-expressing both molecules and no difference among the three groups (figure 4A, online supplemental figure S9A). By day 7, Akt2-OE CTLs rapidly increased perforin and granzyme B expression, while Akt1-OE CTLs reached comparable levels by day 14 (figure 4B,C, online supplemental figure S9A). On ex vivo re-stimulation with the HBc_93-100_ peptide, a small percentage of Akt1-OE and Akt2-OE CTLs secreted IFN-γ at day 4, but Akt2-OE CTLs showed significantly higher IFN-γ and TNF-α production compared with control or Akt1-OE CTLs at days 7 and 14 (figure 4D–F, online supplemental figure S9B). Akt1-OE CTLs displayed delayed cytokine production, with only a small fraction expressing these cytokines by day 14 (figure 4F, online supplemental figure S9B). Control CTLs showed minimal cytotoxic molecule expression or cytokine production during the 4–14 days post-infusion. These findings highlight the superior efficiency of Akt2 signaling in inducing rapid effector functions and enhancing antitumor cytotoxicity compared with Akt1 signaling.

### Differential regulation of immune responses and checkpoint expression by Akt1 and Akt2 signaling in CTLs

**Figure 5:**
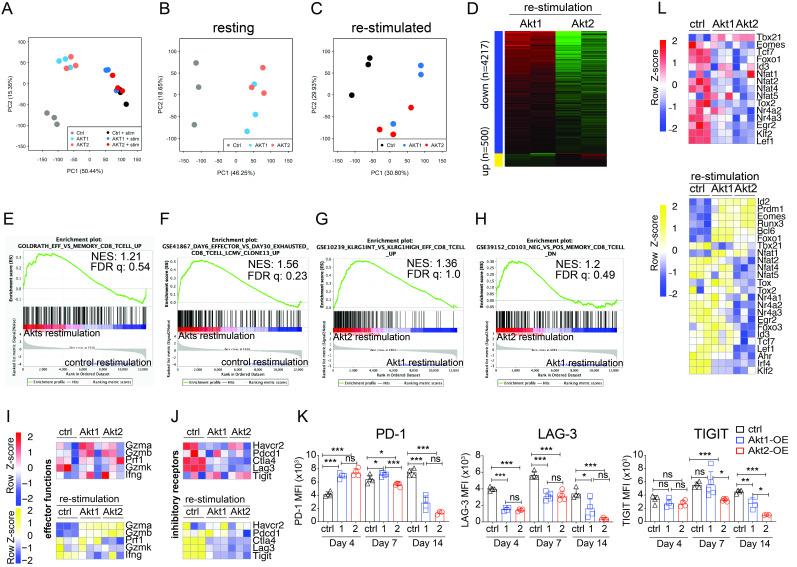
Akt2 overexpression alleviated T-cell exhaustion and promoted tissue resident memory differentiation. (A) Principal component analysis of transcriptomic data from control, Akt1-OE, and Akt2-OE CTLs before (Ctrl, AKT1, AKT2) or after 24-hour restimulation with anti-CD3/anti-CD28 beads, including un-stimulated/resting (B) and restimulated (C) conditions. (D) Heatmap showing 4217 genes downregulated and 500 upregulated genes in Akt2-OE CTLs compared with Akt1-OE CTLs after 24-hour stimulation with anti-CD3/anti-CD28 beads. (E–H) Gene Set Enrichment Analysis of restimulated Akt1-OE and Akt2-OE CTLs compared with control CTLs (E, F) or Akt2-OE versus Akt1-OE CTLs (G, H) using indicated gene sets. (I, J) Heatmaps showing differential expression of genes associated with T-cell effector functions (I) and inhibitory receptors (J) among control, Akt1-OE and Akt2-OE CTLs. (K) Expression levels of immune checkpoints on adoptively transferred CTLs at days 4, 7, and 14 post-transfer. (L) Heatmap showing differential expression of genes associated with T-cell exhaustion and differentiation among control, Akt1-OE and Akt2-OE CTLs. All CTLs analyzed in panels (A–J, L) were collected and analyzed before adoptive transfer. Statistical analysis was performed using unpaired Student’s t-test, and significance is indicated as *p<0.05, **p<0.01, and ***p<0.001. CTLs, cytotoxic T lymphocytes; FDR, false discovery rate; LAG-3, lymphocyte-activation gene 3; MFI, mean fluorescence intensity; NES, normalized enrichment score; PD-1, programmed cell death protein 1; TIGIT, T-cell immunoreceptor with Ig and ITIM domains.

To gain a deeper understanding of the mechanisms underlying how Akt2 signaling enables CTLs to overcome the immunosuppressive HCC TME, we conducted an analysis of global gene expression levels in control CTLs, Akt1-OE CTLs, and Akt2-OE CTLs, both at rest and after re-stimulation, using RNA-seq analysis.^33^ Principal component analysis of the six sets of RNA-seq data revealed higher similarities between resting Akt1-OE and resting Akt2-OE CTLs compared with control CTLs. After 24-hour anti-CD3/anti-CD28 bead restimulation, the gene expression patterns of control, Akt1-OE, and Akt2-OE CTLs tended to become more similar (figure 5A). Further analysis of the three resting CTLs and three re-stimulated CTLs, respectively, unveiled differential gene expression patterns in Akt-OE CTLs compared with control CTLs under resting conditions or after re-stimulation (figure 5B,C). Akt1 or Akt2 signaling also induced slightly diverse gene expression patterns (figure 5B,C). The majority of differentially expressed genes, with at least a onefold change between Akt1-OE CTLs and Akt2-OE CTLs, were upregulated in Akt1-OE CTLs (4,217 genes), with only 500 genes upregulated in Akt2 CTLs (figure 5D). GSEA analysis revealed that both Akt1-OE and Akt2-OE CTLs were enriched in the effector T-cell gene signature of gene set-GOLDRATH_EFF_VS_MEMORY_CD8_TCELL_UP^34^ compared with control CTLs, consistent with the observed decrease in the memory marker CD62L and the high expression of CD44 in Akt-OE CTLs (figures1B  5E). Akt1-OE and Akt2-OE CTLs, compared with control CTLs, were enriched in the effector CD8^+^ T-cell gene signature from gene set GSE41867,^35^ rather than the exhausted CD8^+^ T-cell signature (figure 5F). Further comparison between Akt1-OE and Akt2-OE CTLs revealed that the gene expression profile of Akt2-OE CTLs more closely resembled the KLRG-1-intermediate memory precursor gene signature from GSE10239^36^ or the CD103^+^ tissue-resident memory T-cell gene signature from GSE39152,^37^ consistent with the higher CD69 expression on Akt2-OE CTLs (figures1C  5G,H). RNA-seq data revealed higher expression of granzyme A (*Gzma*) and granzyme B (*Gzmb*) in both Akt1-OE CTLs and Akt2-OE CTLs compared with control CTLs, especially after re-stimulation, although there was less variation in perforin (Prf1), granzyme K (*Gzmk*), and interferon-γ (*Ifng*) messenger RNA (mRNA) expression levels among the three groups (figure 5I). This validates the expression pattern of cytotoxic molecules by the adoptively transferred CTLs, with all three types of CTLs consistently expressing perforin, while only Akt2-OE CTLs exhibited preferential upregulation of granzyme B, contributing to their superior cytotoxic function against cancers (figure 4A–C, online supplemental figure S9A).

Furthermore, the RNA-seq results revealed that Akt1 and Akt2 signaling prevented the transcriptional expression of immune checkpoint molecules *Ctla4*, *Lag3*, and *Tigit* after TCR re-stimulation (figure 5J). The protein expression levels of these immune checkpoints in adoptively transferred CTLs encountering tumors in the TME were more dynamic and varied. Intratumoral Akt1-OE and Akt2-OE CTLs expressed higher levels of PD-1 compared with control CTLs on day 4 post-infusion and gradually reduced PD-1 expression from day 4 to day 14, while control CTLs increased their PD-1 expression during this period (figure 5K, PD-1). Both Akt1-OE and Akt2-OE CTLs showed reduced expression of LAG-3 and TIGIT compared with control CTLs, starting from day 4 post-infusion (figure 5K, LAG-3 and TIGIT). Akt2-OE CTLs maintained lower expression of PD-1 and TIGIT compared with Akt1-OE CTLs at days 7 and 14 (figure 5K, PD-1 and TIGIT).

The regulation of immune checkpoint expression and T-cell exhaustion is typically controlled by T-cell exhaustion-related transcription factors.^38^,^40^ We therefore analyzed the mRNA expression levels of a panel of transcription factors in the three groups of CTLs from in vitro culture. Results showed that Akt1-OE CTLs and Akt2-OE CTLs reduced the mRNA expression of several T-cell exhaustion-related transcription factors, such as *Tox2*, *Nr4a2*, *Nr4a3*, and *Egr2*, compared with control CTLs when they were in a resting state (figure 5L, upper panel). On TCR stimulation, the expression patterns of these transcription factors changed, with Akt2-OE CTLs having lower mRNA levels of *Tox*, *Tox2*, *Nr4a1*, *Nr4a2*, *Nr4a3*, *Egr2*, and *Foxo3* compared with Akt1-OE CTLs or control CTLs (figure 5L, lower panel).

### Akt2 signaling modulates cytosolic calcium levels to prevent NFAT hyperactivation and T-cell exhaustion

**Figure 6:**
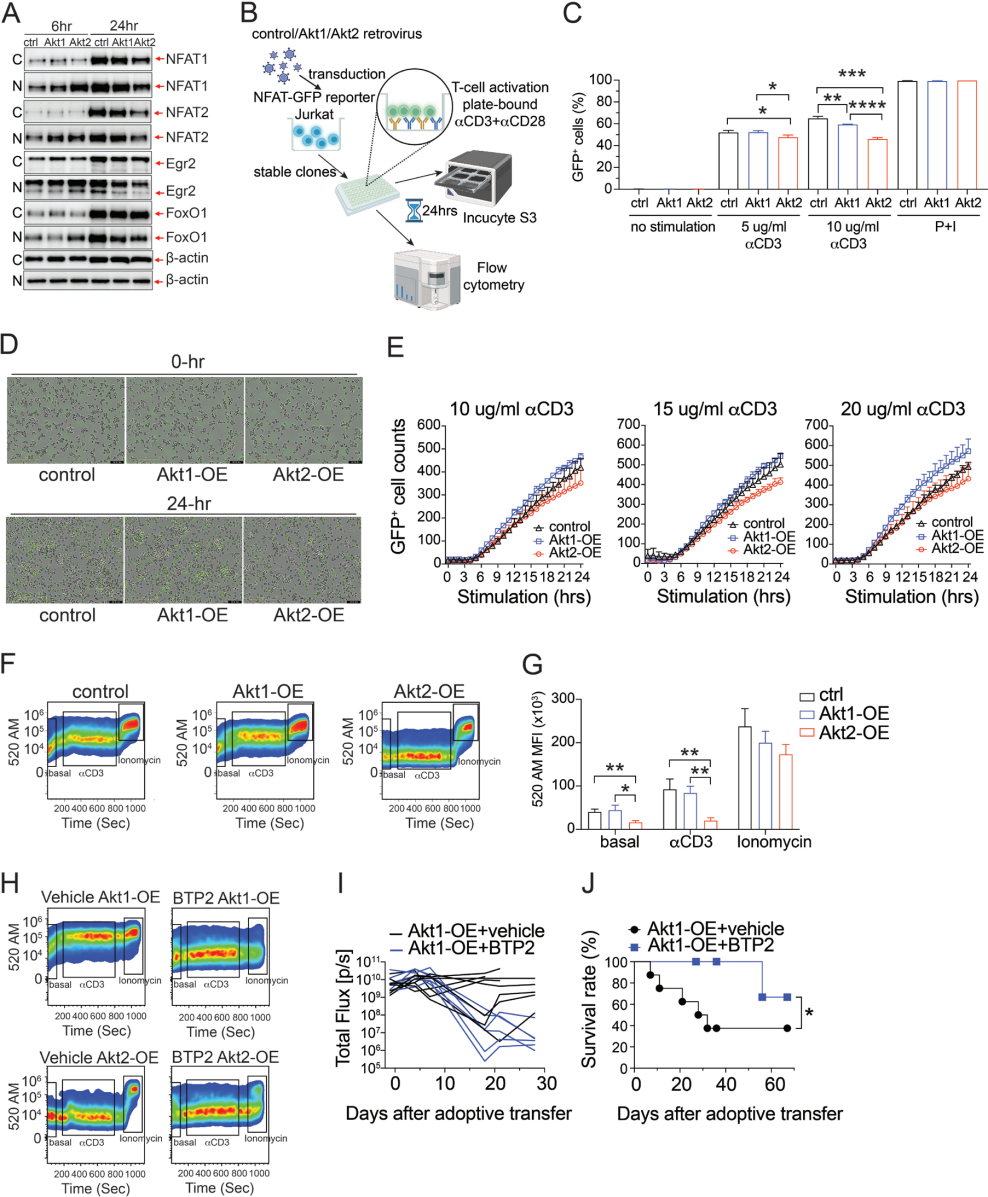
Akt2 signaling prevented T-cell exhaustion of CTLs through regulating calcium influx. (A) Immunoblots showing cytosolic and nuclear levels of NFAT1, NFAT2, Egr2, and FoxO1 in control, Akt1-OE, and Akt2-OE CTLs after 6 or 24 hours of restimulation with anti-CD3/anti-CD28 beads. β-actin was used as a loading control. (B) Schematic of NFAT-GFP reporter assays in Jurkat cells transduced with control, Akt1-OE, or Akt2-OE retroviruses. (C) Percentage of GFP-positive cells in control, Akt1-OE, and Akt2-OE NFAT-GFP reporter Jurkat cells after 24 hours stimulation with anti-CD3/anti-CD28 (n=3). (D) Representative images of GFP expression in NFAT-GFP reporter Jurkat cells at 0 and 24 hours after stimulation. (E) Kinetics of GFP-positive cell counts in NFAT-GFP reporter Jurkat cells stimulated with plate-bound anti-CD3 (10, 15, or 20 µg/mL) and anti-CD28 (1 µg/mL). (F) Intracellular calcium flux kinetics of control, Akt1-OE, and Akt2-OE mouse CTLs after anti-CD3 stimulation followed by ionomycin treatment. (G) Baseline and stimulated intracellular calcium levels in CTLs as in F. (H) Calcium flux kinetics of Akt1-OE and Akt2-OE mouse CTLs treated with vehicle or BTP2 after stimulation with anti-CD3 followed by ionomycin treatment. (I) Tumor growth kinetics in HCC-bearing mice receiving 1×10^6^ vehicle-treated (n=8) or BTP2-treated (n=6) Akt1-OE CTLs. (J) Survival curves of HCC-bearing mice from I. Statistical analysis was performed using unpaired Student’s t-test or Kaplan-Meier analysis (J), and significance is indicated as *p<0.05, **p<0.01, and ***p<0.001, ****p<0.0001. CTLs, cytotoxic T lymphocytes; GFP, green fluorescent protein; HCC, hepatocellular carcinoma; NFAT, nuclear factor of activated T cells.

From the RNA-seq data, we observed notable differences in the mRNA levels of genes belonging to the NFAT family between Akt2-OE CTLs and Akt1-OE CTLs following restimulation (figure 5L, lower panel). NFAT signaling serves a pivotal role in T-cell activation on TCR triggering, but it becomes involved in T-cell exhaustion when persistent antigen stimulation occurs. Consequently, we conducted an analysis of the protein levels of NFAT1 and NFAT2 in these CTLs at various time points following TCR restimulation through western blotting. In the initial stages of TCR activation (6 hours), most of the NFAT1 and NFAT2 proteins were found inside the cell nucleus rather than in the cytoplasm of CTLs and both Akt1-OE and Akt2-OE CTLs showed an increased expression of NFAT1 and NFAT2 when compared with control CTLs (figure 6A, online supplemental figure S10). However, as the time period progressed from 6 hours to 24 hours following TCR re-stimulation, both control and Akt1-OE CTLs continued to elevate the levels of nuclear NFAT1 and NFAT2. Conversely, Akt2-OE CTLs exhibited a decline in the nuclear levels of NFAT1 and NFAT2 during this same period (figure 6A, online supplemental figure S10). The NFAT signaling pathway plays a critical role in regulating the expression of transcription factors associated with T-cell exhaustion. This includes Egr2, which acts as a potent activator of LAG-3 expression.^41^ Our observations indicate that Akt signaling, particularly Akt2, led to a reduction in both the nuclear protein and mRNA levels of Egr2 (figures6A  5L) from 6 hours to 24 hours post-re-stimulation. This provides an explanation for the prevention of LAG-3 expression observed in CTLs overexpressing Akt1 and Akt2 (figure 5K). Additionally, Akt signaling also resulted in decreased nuclear expression of FoxO1, a crucial transcription factor involved in the regulation of PD-1 transcription, from 6 hours to 24 hours post-re-stimulation (figure 6A, online supplemental figure S10). To further prove that Akt2 signaling suppresses NFAT transcriptional activity to prevent T-cell exhaustion, we established three stable cell lines expressing CD90.1 (control Jurkat), Akt1-CD90.1 (Akt1-OE Jurkat), and Akt2-CD90.1 (Akt2-OE Jurkat) based on an NFAT-GFP reporter Jurkat cell line (figure 6B). Before stimulation, all three cell lines were negative for GFP expression as detected by flow cytometry (figure 6C, no stimulation). Using ionomycin (a Ca^2+^ ionophore) and phorbol 12-myristate 13-acetate (PMA, a protein kinase C activator) as positive controls for the NFAT reporter assay, we found that nearly all cells became GFP-positive after stimulation with PMA and ionomycin (figure 6C, P+I). When using plate-bound anti-CD3 and anti-CD28 antibodies, fewer Akt2-OE Jurkat cells turned GFP-positive compared with control or Akt1-OE Jurkat cells under two different stimulation conditions (figure 6C). We used the IncuCyte live cell imaging system to detect the kinetics of NFAT transcriptional activity under anti-CD3 and anti-CD28 stimulation (figure 6B). The results revealed that Akt1 signaling enhanced NFAT transcriptional activity, whereas Akt2 signaling inhibited it under three different stimulation conditions (figure 6D,E).

Since NFAT activation during T-cell activation is highly dependent on the cytosolic Ca^2+^ level, we postulate that Akt2 signaling may regulate the calcium influx on TCR triggering. We labelled the primary mouse control CTLs, Akt1-OE CTLs, and Akt2-OE CTLs, as well as control Jurkat, Akt1-OE Jurkat, and Akt2-OE Jurkat cells, with a calcium-sensitive dye, Cal-520 AM. Additionally, we stained the cells with antibodies specific for CD90.1 (a transgene expression reporter) and mouse CD8α (only for mouse primary CTLs) and analyzed cytosolic calcium levels using flow cytometry under each indicated condition (online supplemental figure S1). We observed that Akt2-OE CTLs had a lower basal level of intracellular Ca^2+^ compared to control CTLs and Akt1-CTLs (figure 6F,G). Following TCR stimulation, cytosolic Ca^2+^ levels increased in control CTLs or Akt1-OE CTLs but remained low in Akt2-OE CTLs, even though the maximum calcium import capacity of Akt2-OE CTLs induced by ionomycin was similar to the other two CTL types (figure 6F,G). Akt2 overexpression also led to a reduction in intracellular Ca^2+^ level in Akt2-OE Jurkat cells when compared with Akt1-OE Jurkat cells (online supplemental figure S11). These findings strongly suggest that Akt2 signaling plays a critical role in regulating NFAT activation through modulating the influx of calcium ions and also provide a plausible explanation for why Akt2-OE CTLs exhibit resistance to T-cell exhaustion and persist in the HCC TME.

We further investigated whether continuous calcium signaling in Akt1-OE CTLs was responsible for their inability to effectively combat liver cancers. Both Akt1-OE and Akt2-OE CTLs were treated with the store-operated calcium channel inhibitor, BTP2, for a duration of 24 hours before adoptive transfer. We observed that the BTP2 treatment could block the calcium influx in Akt1-OE CTLs to the level observed in Akt2-OE CTLs following TCR activation (figure 6H). We then adoptively transferred the vehicle-treated or the BTP2-treated Akt1-OE CTLs into HCC-bearing mice. Interestingly, the BTP2-treated Akt1-OE CTLs exhibited a more efficient elimination of HCC compared with their vehicle-treated counterparts (figure 6I, online supplemental figure S12). Mice that received the vehicle-treated Akt1-OE CTLs had a median survival period of 30 days, while those receiving the BTP2-treated Akt1-OE CTLs significantly extended their median survival to over 60 days (figure 6J, online supplemental figure S12).

We have demonstrated that Akt2 signaling plays a role in regulating calcium mobilization, thus influencing NFAT activation and preventing T-cell exhaustion in primary mouse CTLs and in a human T-cell line. Whether a similar mechanism also operates in primary human CD8^+^ T cells requires further investigation.

**Figure 7:**
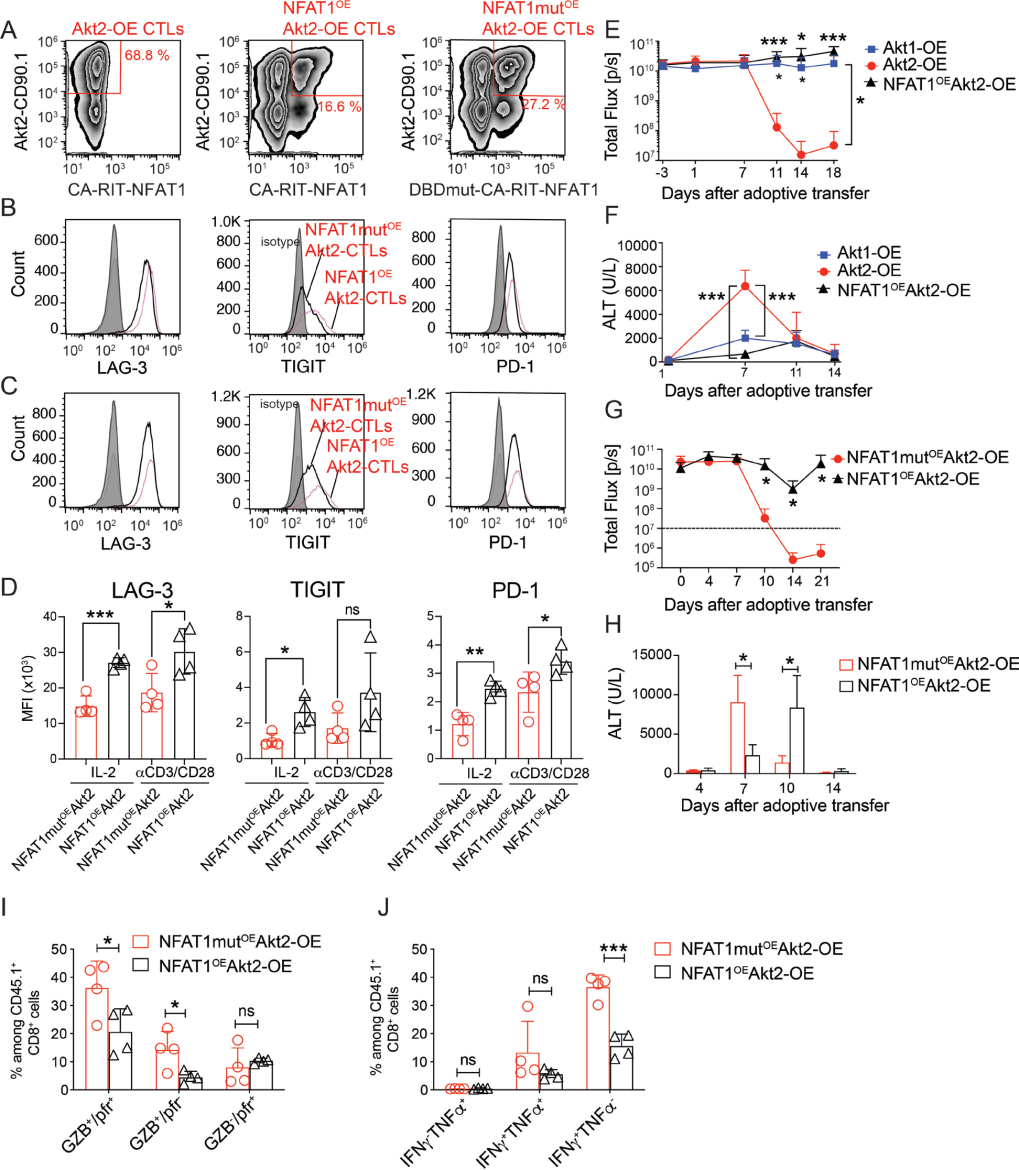
NFAT1 reactivation dampened the antitumor capability of Akt2-OE CTLs. (A) Gating strategies of Akt2-OE CTLs (left), NFAT1^OE^Akt2-OE CTLs (middle) and NFAT1mut^OE^Akt2-OE CTLs (right). (B, C) Histograms showing LAG-3, TIGIT, and PD-1 expression on indicated CTLs cultured with interleukin-2 (50 IU/mL) for 24 hours (B) or restimulated with anti-CD3/anti-CD28 beads for 24 hours (C). (D) MFI of LAG-3, TIGIT, and PD-1 expression on CTLs from B to C. (E, F) Tumor burden (total flux) (E) and serum ALT levels (F) in HCC-bearing mice receiving 4×10^5^ FACSorted Akt1-OE (n=4), Akt2-OE (n=7) or NFAT1^OE^Akt2-OE CTLs (n=3). (G, H) Tumor burden (G) (total flux) and serum ALT levels (H) in HCC-bearing mice receiving 4×10^5^ FACSorted NFAT1^OE^Akt2-OE CTLs or NFAT1mut^OE^Akt2-OE CTLs. (n=4). (I) Percentage of perforin and granzyme B expressing cells among CD45.1^+^ CTLs isolated from the tumor masses of HCC-bearing mice receiving 4×10^5^ FACSorted NFAT1^OE^Akt2-OE CTLs or NFAT1mut^OE^Akt2-OE CTLs at day 7. (J) Percentage of cytokine-producing cells among CD45.1^+^ CTLs isolated from the tumor masses of HCC-bearing mice as described in I. Statistical analysis was performed using unpaired Student’s t-test, and significance is indicated as *p<0.05, **p<0.01, and ***p<0.001. ALT, alanine aminotransferase; CTLs, cytotoxic T lymphocytes; HCC, hepatocellular carcinoma; IFN, interferon; LAG-3, lymphocyte-activation gene 3; MFI, mean fluorescence intensity; NFAT, nuclear factor of activated T cells; PD-1, programmed cell death protein 1; TIGIT, T-cell immunoreceptor with Ig and ITIM domains; TNF, tumor necrosis factor.

We further manifested whether the suppression of NFAT activation is a crucial mechanism in Akt2-mediated antitumor functionality. In addition to Akt2 overexpression, we engineered mouse CTLs using retroviruses encoding constitutively active Nfat1 (CA-RIT-NFAT1) or a mutant incapable of binding to DNA (DBDmut-CA-RIT-NFAT1), as previously described.^38^ The reporter proteins used were CD90.1 for Akt expression and GFP for NFAT1 expression. Flow cytometry analysis revealed the expression of Akt2-CD90.1 and NFAT1-GFP on engineered CTLs (figure 7A: left panel for Akt2 transduction, central panel for Akt2+NFAT1 co-transduction, right panel for Akt2+NFAT1 mutant co-transduction).

We measured the expression of immune checkpoints on resting CTLs incubated in the presence of interleukin (IL)-2 or on CTLs on restimulation for 24 hours. NFAT1^OE^Akt2-OE CTLs (CD90.1^+^ GFP^+^) exhibited minimal or no differences in the levels of LAG-3, TIGIT, and PD-1 compared to NFAT1mut^OE^Akt2-OE CTLs in the resting state (figure 7B,D, IL-2) or on restimulation (figure 7C,D, αCD3/αCD28). We sorted engineered CD90.1^+^CTLs (Akt1-OE or Akt2-OE CTLs) or CD90.1^+^GFP^+^ CTLs (NFAT1^OE^Akt2-OE CTLs) from the in vitro CTL culture and adoptively transferred them into HCC-bearing mice. Akt2-OE CTLs efficiently eliminated HCC within 14 days, as expected. However, NFAT1^OE^Akt2-OE CTLs, like Akt1-OE CTLs, failed to eliminate HCC (figure 7E, online supplemental figure S13). NFAT1^OE^Akt2-OE CTLs exhibited delayed cytotoxicity, as indicated by lower serum ALT levels compared with Akt2-OE CTLs at day 7 post-infusion (figure 7F). In another independent experiment, it was observed that NFAT1mut^OE^Akt2-OE CTLs, like Akt2-OE CTLs displayed a notable ability to rapidly initiate cytotoxic responses and effectively eliminate HCC (figure 7G,H, online supplemental figure S14). Furthermore, NFAT1^OE^Akt2-OE CTLs demonstrated significantly reduced effector functions, with lower granzyme B and perforin expression and diminished IFN-γ and TNF-α production compared with NFAT1mut^OE^Akt2-OE CTLs (figure 7I,J, online supplemental figure S15). These results suggest that NFAT1 overexpression impairs the effector functions of Akt2-OE CTLs, highlighting the critical balance between NFAT activity and cytotoxic potential in maintaining effective T-cell responses against HCC.

These findings collectively illustrate that both Akt1 and Akt2 overexpression enhance the functional capabilities of mouse CTLs, although with different dynamics. Akt2 signaling, in particular, accelerates these effector functions for a rapid attack on tumor cells. Concurrently, Akt1 signaling, as anticipated, triggers calcium influx and subsequent NFAT activation, whereas Akt2 signaling curbs calcium influx, preventing excessive NFAT expression and nuclear translocation. This inhibition of NFAT’s transcriptional activity by Akt2 signaling during prolonged antigen stimulation subsequently leads to reduced expression of transcription factors associated with T-cell exhaustion, such as Egr2, Nr4a2, Tox, and immune checkpoints. Consequently, this prevents T-cell exhaustion within the TME and results in efficient tumor eradication.

## Discussion

The PI3K/Akt signaling pathway plays a crucial role in inducing the effector function of CTLs, particularly in activating CD8^+^ T cells and promoting the expression of cytotoxic molecules.^1 42^ On phosphorylation and activation, Akt moves from the membrane to the cytosol and nucleus and then phosphorylates FoxO1/2 transcription factors, leading to their degradation. This inhibits the expression of homing adhesion molecules such as CD62L and CCR7, transcriptionally regulated by FoxO1, thereby preventing the differentiation of central memory T cells.^43 44^ Additionally, activated Akt phosphorylates and inactivates GSK3β, which stabilizes and relocates NFATs to the nucleus.^45^ Translocated NFAT proteins, along with other transcription factors like AP1, FOXP3, and BATF, regulate gene expression controlling cytokine production, cytotoxicity, or T-cell differentiation.^46 47^ However, NFAT proteins, especially NFAT1, NFAT2, and NFAT4, have been implicated in T-cell exhaustion when not partnered with other transcriptional factors.^38 40 48^ Although NFAT5 differs structurally and functionally from other isoforms, recent evidence suggests its involvement in regulating T-cell exhaustion in the TME, but not in chronic viral infection.^49^ There is evidence showing that the PI3K/Akt/mTOR pathway has a great influence on epigenetic reprogramming through regulation of DNA methylation^50^,^53^ and histone modifications.^53^,^55^ Our findings indicate that Akt2 activation in CTLs reduces the expression of NFATs, particularly NFAT2, NFAT4, and NFAT5. It would be intriguing to investigate whether Akt1 and Akt2 differentially affect epigenetic machinery such as DNA methyltransferases, histone acetylases, or ten-eleven translocation methylcytosine dioxygenases to regulate the expression of genes related to T-cell exhaustion.

We observed differential antitumoral abilities between Akt2-OE CTLs and Akt1-OE CTLs, with no variance in their antiviral capabilities. In this HBV mouse model, HBV virions produced from the AdHBV infection in hepatocytes were unable to infect surrounding hepatocytes due to the limitation of host range,^26^ confirming the strong intrahepatic cytotoxic capability of Akt1-OE CTLs on one-round Ag stimulation. However, in the HCC mouse model,^27^ Akt1-OE CTLs exhibited delayed effector function compared with Akt2-OE CTLs and failed to eliminate tumor cells initially. As tumor cells proliferated rapidly, the tumor Ag load increased, repeatedly stimulating Akt1-OE CTLs and triggering NFAT pathway activation. NFAT activation in immune cells is typically regulated by cytosolic Ca^2+^ levels enhanced by store-operated Ca^2+^ entry through activated CRAC channels.^56^ Short-duration Ca^2+^ signal transduction rapidly influences T-cell motility and degranulation,^57 58^ while long-duration types are associated with new gene expression, regulating lymphocyte proliferation, cytokine or chemokine production, T-cell differentiation, and anergy.^56^

On reactivation, both Akt1 and Akt2 signaling rapidly enhance NFAT1 and NFAT2 nuclear translocation, boosting the effector functions of Akt1-OE and Akt2-OE CTLs in vivo and in vitro (data not shown). This suggests that both efficiently trigger short-duration Ca^2+^ signaling, leading to NFAT activation, CTL degranulation, and tumor cell killing. However, only Akt2-OE CTLs, not Akt1-OE CTLs, effectively eliminate tumor cells in vivo, accompanied by distinct exhaustion-related gene expression. We speculate that Akt2, but not Akt1, prevents T-cell exhaustion in the HCC TME by suppressing long-duration Ca²^+^ signals, consistent with prior findings showing that Akt2 regulates calcium mobilization and NFAT activation by limiting ER calcium release.^24^ Previous studies have demonstrated that inhibiting calcium signaling can enhance the function of human chimeric Ag receptor (CAR)-T cells.^59^ While our data are derived from mouse CTLs and a human T-cell line, further studies are needed to confirm whether Akt2 exerts similar regulation on calcium signaling and NFAT activation in primary human CD8^+^ T cells, which is essential for its application in human T-cell therapy.

Recently, T-cell-based immunotherapy has garnered significant attention in the field of cancer treatment, particularly with the emergence of CAR-T technology. While CAR-T therapy has shown promising therapeutic efficacy in hematological malignancies, there remains ample room for improvement in its effectiveness for treating solid tumors.^60 61^ The dysfunction of effector immune cells, including CAR-T cells, within solid tumors is attributed to various factors such as poor cell trafficking and tumor infiltration, immunosuppressive cell populations, and tumor-derived suppressive factors, all of which contribute to T-cell exhaustion. Strategies aimed at alleviating T-cell suppression, including the use of soluble decoys and single-chain variable fragments targeting immune checkpoints, dominant negative receptor decoys, knockdown or knockout of inhibitory genes, and interference with inhibitor signaling, are actively under development.^62^ Transcription factors that regulate T-cell activation, differentiation, and T-cell exhaustion have also been shown to significantly influence the antitumor efficacy of CAR-T cells in the treatment of solid tumors.^3963^,^66^

It has been suggested that inhibition of Akt signaling during the initial CAR-T cell preparation helps to maintain a T-cell memory phenotype and enhance the in vivo antitumor efficacy of CAR-T cells.^67 68^ However, the allosteric Akt inhibitor MK-2206, used in the study, only transiently suppresses Akt activation/phosphorylation, and phosphorylated Akt reappears in CAR-T cells before they are adoptively transferred to tumor-bearing mice; thus, the real status of Akt activation in CTLs within the TME is not addressed.^68^ Akt activation by PDK-1 is crucial for effector-like memory CD8^+^ T-cell development and tumor immune surveillance.^8^ Previous studies have shown differential effects on CD44 and CD62L expression in Akt1 and Akt2 knockout CD8^+^ T cells, with Akt1 knockout CD8^+^ T cells showing a higher proportion of CD44^+^CD62L^+^ memory cells, whereas Akt2 knockout CD8^+^ T cells exhibited a proportion of CD44^+^CD62L^+^ memory cells only slightly higher than that of wild-type CD8^+^ T cells, suggesting Akt1 primarily drives terminal differentiation of CD8^+^ T cells.^23^

Under normal conditions, T cells predominantly express Akt1, crucial for TCR signaling and activation,^31^ while Akt2 is mainly expressed in non-immune tissues like the liver and muscle, regulating glucose homeostasis.^69^ In our study, ectopic expression of Akt1 and Akt2 resulted in similar protein levels, but Akt2 exhibited significantly higher T308 and S473 phosphorylation, potentially due to its regulation of glucose homeostasis affecting PI3K and mTORC2 activity, enhancing Akt phosphorylation and T-cell effector functions. Akt3, primarily expressed in the brain and testes, showed minimal relevance in T cells, lacking T308 phosphorylation but activating downstream targets like S6 kinase. This suggests Akt3 may regulate T cells through alternative mechanisms, including potential negative feedback loops that attenuate PI3K activity and Akt phosphorylation,^70^ warranting further investigation.

Our study clearly demonstrates that although both Akt1 and Akt2 signaling can induce CTL differentiation toward the effector stage and promote their effector functions, only Akt2 signaling enables CTLs to resist the immunosuppressive TME due to its ability to induce prompt cytotoxicity and suppress long-duration calcium influx. These findings highlight the isoform-specific roles of Akt signaling in T-cell differentiation and function, emphasizing the need to carefully consider the activation or inhibition of specific Akt isoforms in therapeutic applications. Further studies focusing on Akt2 signaling in CD8^+^ T-cell differentiation could uncover additional mechanisms that enhance T-cell persistence, memory potential, and antitumor efficacy, thereby broadening its therapeutic applicability in cancer immunotherapy.

## supplementary material

10.1136/jitc-2024-009827 online supplemental file 1

## Data Availability

Data are available in a public, open access repository.
