# Supplementary material for: Differential regulation of calcium-NFAT signaling pathway by Akt isoforms: unraveling effector dynamics and exhaustion of cytotoxic T lymphocytes in tumor microenvironment
Source: J Immunother Cancer. 2025 Mar 26;13(3):e009827. doi: 10.1136/jitc-2024-009827 (PMC11951004; doi:10.1136/jitc-2024-009827)
Supplement: online supplemental file 1 [file jitc-13-3-s001.pdf]

Differential regulation of calcium-NFAT signaling pathway by Akt isoforms: unraveling effector dynamics and exhaustion of cytotoxic T lymphocytes in tumor microenvironment

Wen-Ling Chen[1,*], Yong-Lin Chang[1,*], Su-Fang Lin[2], Ulrike Protzer[3], Masanori Isogawa[4], Hung-Chih Yang[5, 6, 7], and Li-Rung Huang[1,7]

[1]Institute of Molecular and Genomic Medicine, National Health Research Institutes, Miaoli, Taiwan

[2]National Institute of Cancer Research, National Health Research Institutes, Miaoli, Taiwan

[3]Institute of Virology, School of Medicine and Health, Technical University of Munich/ Helmholtz Munich, Germany.

[4]Department of Virology II, National Institute of Infectious Diseases, Tokyo, Japan.

[5]Division of Gastroenterology and Hepatology, Department of Internal Medicine, National Taiwan University Hospital, Taipei, Taiwan

[6]Graduate Institute of Microbiology, National Taiwan University, Taipei, Taiwan

[7]Graduate Institute of Clinical Medicine, National Taiwan University, Taipei, Taiwan

[*]These authors contributed to the work equally.

Correspondence: Dr. Li-Rung Huang, Institute of Molecular and Genomic Medicine, National Health Research Institutes, No.35, Keyan Road, Zhunan town, Miaoli County 350, Taiwan; Tel: +886 (0)37 206166 ext. 35346; E-mail: lrhuang@nhri.edu.tw

Supplemental methods

Oncogene-induced HCC mouse model

To induce hepatocellular carcinoma (HCC), C57BL/6J mice were anesthetized with Isoflurane mixed with O_2 before receiving hydrodynamic injections (HDI) of endotoxin-free plasmids dissolved in Dulbecco's Phosphate Buffered Saline (DPBS) equivalent to 8% of body weight within 5 seconds. Each mouse received 2 μg of pCMV(CAT)T7-SB100, 10 μg of pT/Caggs-NRASV12, and 10 μg of pKT2/CLP-AKT-2A-OVA-HBc-HBs-LUC plasmids. Photon emission from transduced hepatocytes or tumor cells was monitored periodically using an IVIS imaging system (Caliper Life Sciences, Massachusetts, USA). HCC-bearing mice with a total photon flux from IVIS imaging exceeding $1x10^8$ photons/sec were grouped evenly and used as recipients for adoptive T-cell transfer, while those with a total flux exceeding $3x10^{10}$ photons/sec or experienced a ≥20% weight loss were humanely euthanized to prevent suffering from large liver tumors.

Grouping method and sample size

To allocate experimental units to control and treatment groups, we employed a systematic allocation strategy based on baseline measurements. For HCC-bearing mice, animals were ranked from highest to lowest bioluminescent signal intensity and sequentially assigned to groups following a predetermined sequence (Group 1, 2, 3, 3, 2, 1, 1, 2, 3, 3, 2, 1, …). Similarly, for HBV carrier, animals were ranked by serum HBeAg levels before allocation using the same method. This approach ensured that each group had a balanced distribution of initial conditions while

minimizing variability between groups.
The sample size for animal experiments was determined based on statistical power analysis, prior literature, and experimental feasibility. To ensure that our study could detect biologically meaningful differences with sufficient statistical power, we designed experiments with n = 4–9 per group, depending on the specific assay and expected effect size. Power calculations were conducted to achieve at least 80% power at a significance level of $P < 0.05$, ensuring the ability to detect relevant differences in immune responses, tumor burden, and T cell function. The exact numbers of mice in each group of every experiment were indicated in figure legends or directly be shown in the figures.
Total mice number used in each experiment.
Figure 1: 18 recipient mice
Figure 2: 67 recipient mice
Figure 3: 68 recipient mice
Figure 4: 39 recipient mice
Figure 5: 3 mice
Figure 6: 14 recipient mice
Figure 7: 30 recipient mice
There were around 350 mice used in this study including those without HBeAg persistence or HCC growth after induction.

Cell isolation and flow cytometric analysis
Spleens, draining lymph nodes, and liver/tumors were harvested from mice at specified time points. Liver/tumor tissues underwent perfusion via the portal vein with a digestion solution containing 0.5 mg/ml collagenase IA (Sigma-Aldrich, St. Louis, USA), followed by mechanical disruption and digestion for 30 minutes at 37°C in Gey's balanced salt solution (GBSS) supplemented with 0.5 mg/ml collagenase IA. The resulting liver/tumor cell homogenate was suspended in 4 mL of 40% Percoll gradient density media (GE Healthcare Life Sciences) and layered over 4 mL of 80% Percoll for gradient centrifugation at 1400 g for 20 minutes at room temperature. Leukocytes were collected from the interface and used for surface marker or intracellular staining followed by flow cytometric analysis. Spleens and lymph nodes were mechanically disrupted to obtain single-cell suspensions for further analysis. Staining procedures were conducted in the presence of 10 μg/mL of Fc receptor block (clone: 2.4G2) in fluorescence-activated cell sorting buffer (FACS buffer, phosphate-buffered saline/2% bovine serum albumin/0.02% NaN_3). Data acquisition and analysis were performed using an Attune NxT flow cytometer (Thermo Fisher Scientific) or Cytek Aurora (Cytek Biosciences) and analyzed with FlowJo software (V.10.0.8r1, FlowJo, LLC, Ashland, USA). Antibodies and dyes used for surface marker or intracellular staining are listed in Table S1.

Immunohistochemistry study
Paraffin-embedded liver/tumor tissue sections were deparaffinized, rehydrated, followed by heat-induced antigen retrieval and then incubated with primary antibodies as listed in table S1.

ImmPRESS anti-Rat Ig, ImmPRESS anti-Rabbit Ig polymer detection kits, DAB peroxidase substrate kit and hematoxylin (all from Vector laboratories, Burlingame, USA) were used for detection and visualization. For detection of $CD45.1^+$ adoptively transferred $CD8^+$ T cells, 5-7 μm of liver/tumor tissue frozen sections were fixed in ice cold acetone/chloroform (1:1), incubated with FITC-conjugated anti-CD45.1 (A20, Biolegend) and anti-FITC conjugate HRP (Thermo Fisher Scientific), followed by incubation with DAB substrate. The images were captured using an automatic digital slide scanner Pannoramic MIDI with Plan-Apochromat 20x/0.8 objective (3D HISTECH).

CTL preparation for RNA purification for RNA-seq

The activated HBc_{93-100}-specific $CD8^+$ T cells were transduced with retroviruses carrying the control CD90.1, Akt1-CD90.1 or Akt2-CD90.1 ORFs at day 1 post activation. The activated $CD8^+$ T cells were harvested at day 3 post activation and were re-stimulated with anti-CD3/anti-CD28 beads for 24 hours. The transduced $CD8^+$ T cells at day 3 post activation or under re-stimulation for 24 hours were FACSorted based on CD90.1 expression and were subjected to RNA extraction using TRIzol reagent (Thermo Fisher Scientific).

Figure S1

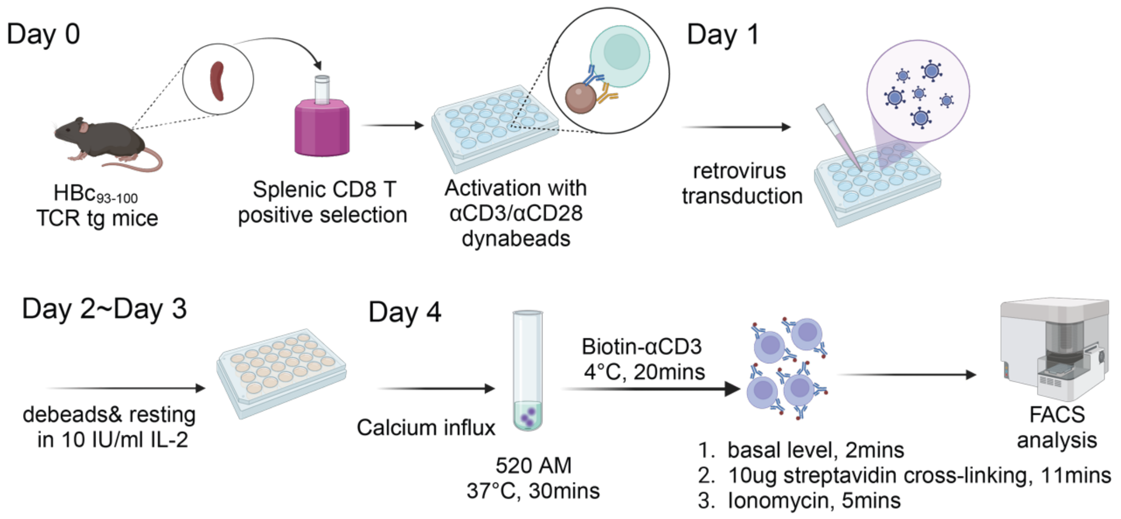

Figure S1. Flow chart of monitoring calcium mobilization in primary mouse CTLs.

Figure S2

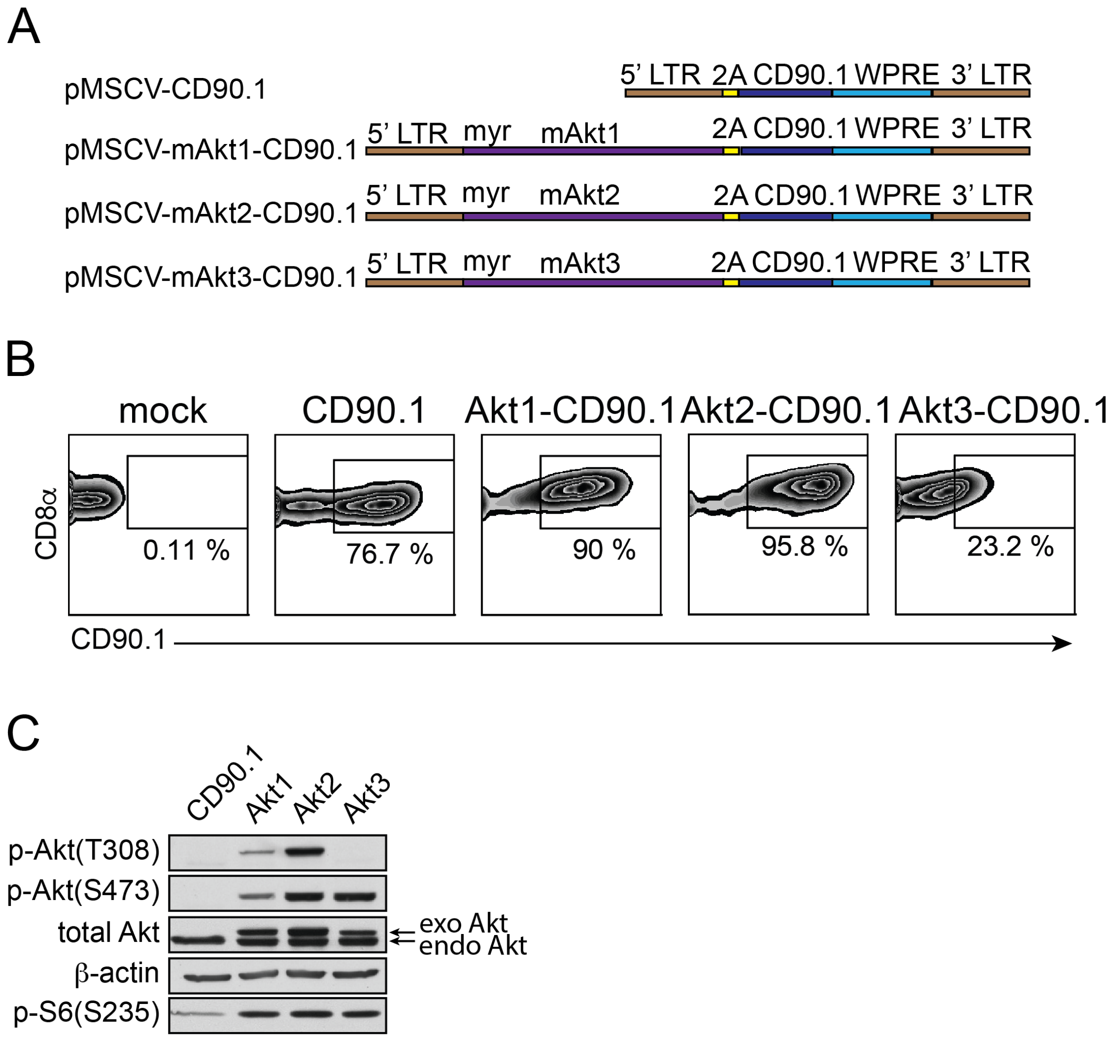

Figure S2. Retroviral constructs and protein expression after T-cell engineering. (**A**) Schematic representation of MSCV retroviral constructs used for T-cell engineering. (**B**) Transduction efficiency of *in vitro*-activated CD45.1^{+}OT-I cells transduced with retroviruses carrying 2A-CD90.1, mAkt1-2A-CD90.1, mAkt2-2A-CD90.1 and mAkt3-2A-CD90.1, respectively or mock. At day 2 after transduction, the surface expression of CD90.1 as a marker for successful transduction was detected by flow cytometric analysis. (**C**) Western blot for detection of phospho-Akt, total Akt, β-actin and phospho-S6 proteins in the cell lysate of ctrl, Akt1, Akt2 and Akt3-OE CD8^{+} T cells.

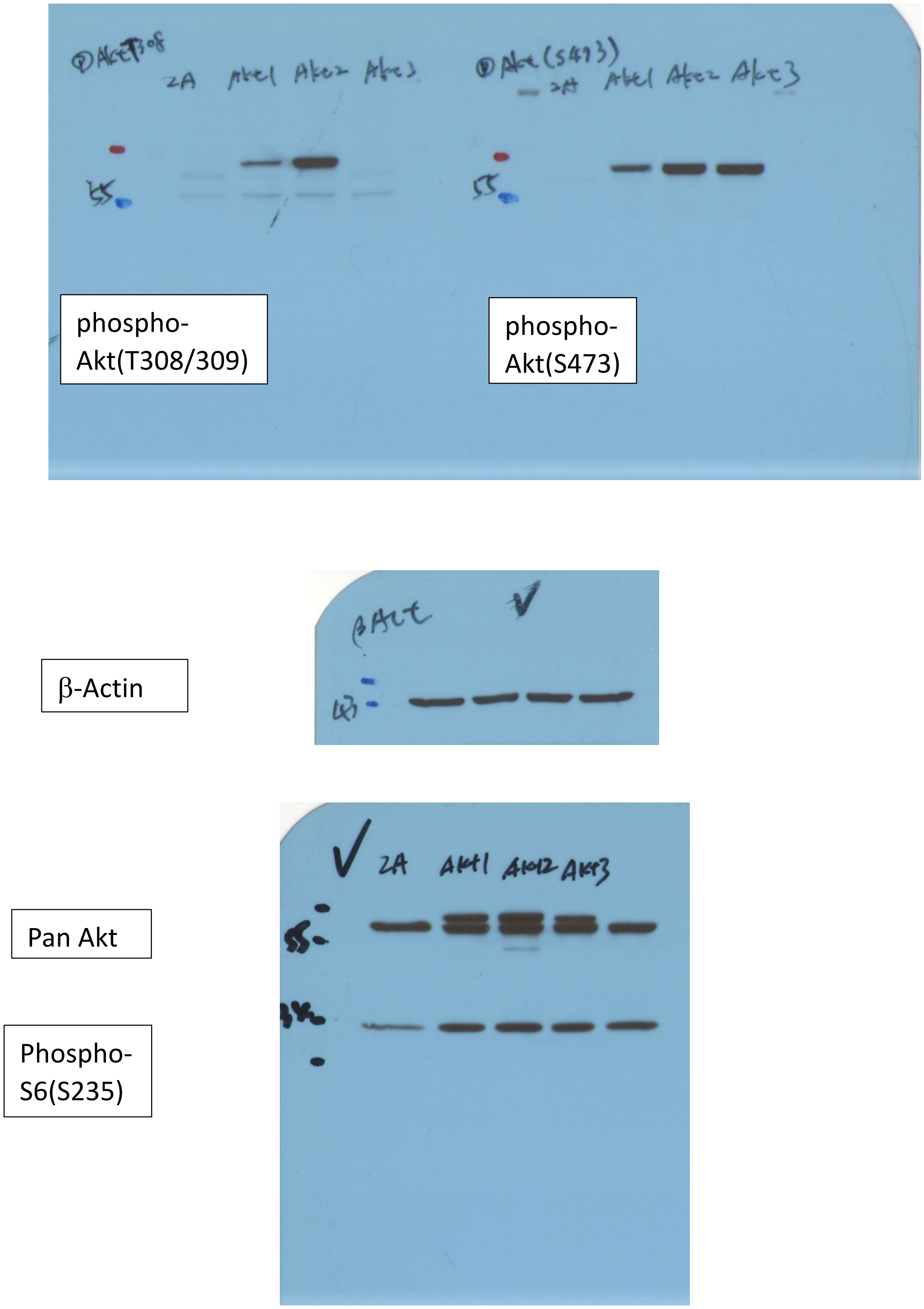

Figure S3. The uncut raw Western blot imaging of Figure S2C

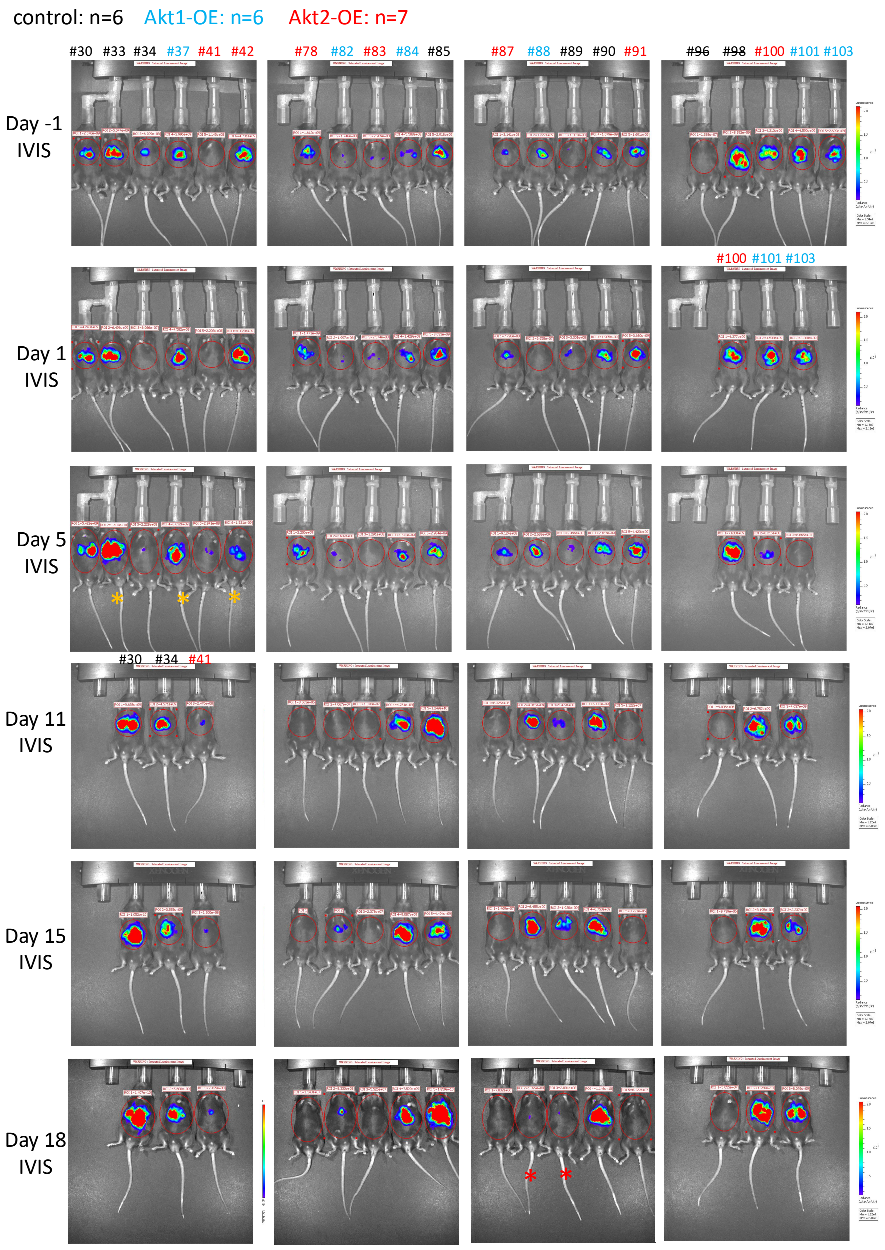

Figure S4. Tumor progression monitored by IVIS imaging in mice undergoing adoptive T-cell therapy. IVIS imaging was performed at multiple time points (Day -1, Day 1, Day 5, Day 11, Day 15, and Day 18) to evaluate tumor progression in mice treated with adoptive T-cell therapy. Mice were stratified into three groups, Akt1-OE (blue mouse ID), Akt2-OE (red mouse ID), and control (black mouse ID), (n = 6–7 per group) based on their IVIS bioluminescence values at Day -1, ensuring comparable initial tumor burden across groups. On day 0, mice in the treatment groups were administered $2x10^5$ engineered CTLs. All images are shown for each time point, illustrating tumor burden differences among groups.

The yellow-starred (*) mice at Day 5 were sacrificed due to illness after treatment. The red-starred mice (*) exhibited discrepancies in IVIS signals, likely caused by tumor size interfering with luciferin absorption, resulting in inconsistencies between IVIS values and the liver tumor size observed at d19 necropsy. This data is summarized in the line-dot plot presented in **Figure 2B**, providing a quantitative representation of tumor progression over time.

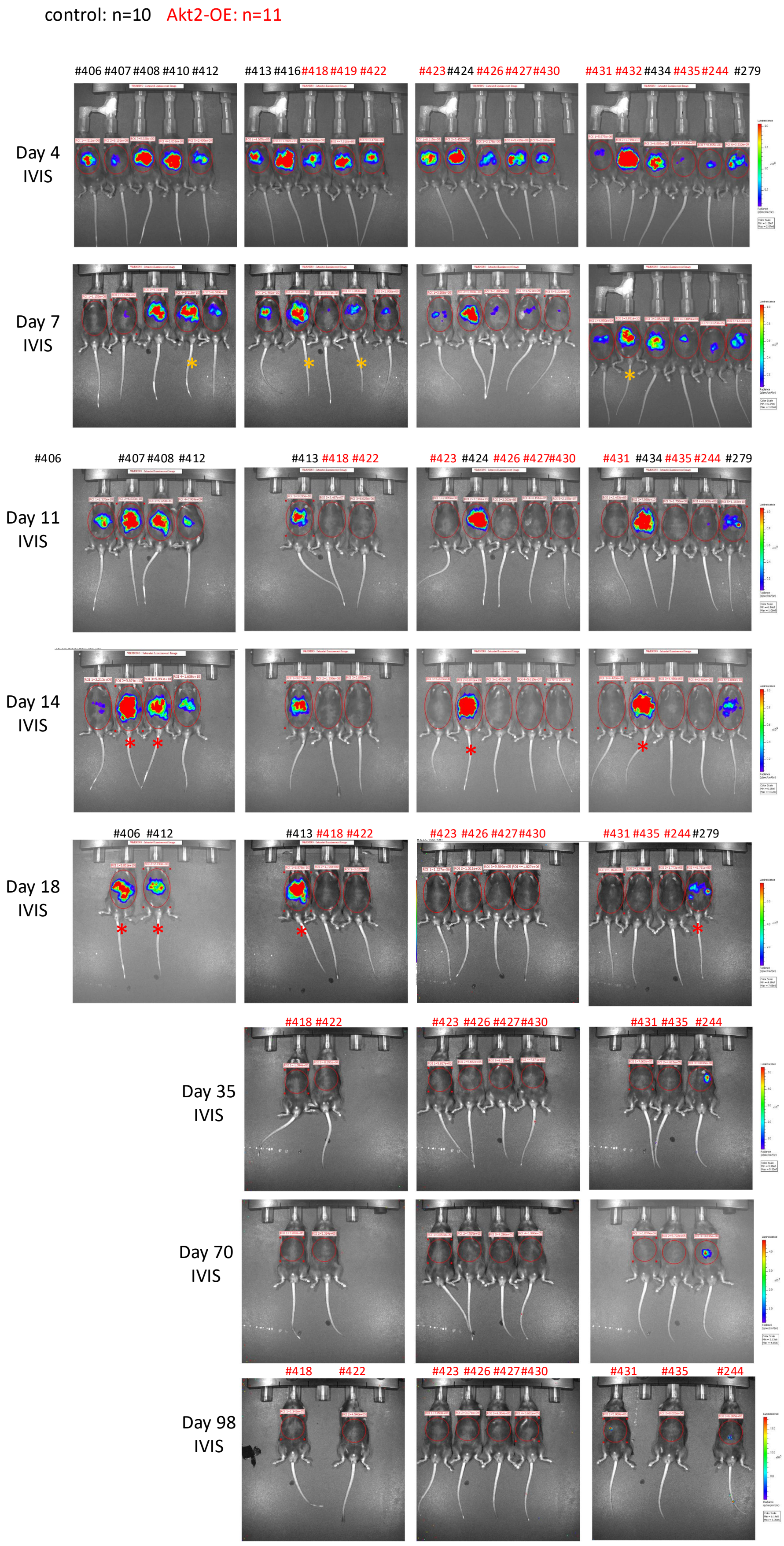

Figure S5. Longitudinal monitoring of tumor suppression following adoptive T-cell transfer therapy. IVIS imaging was conducted at multiple time points (Day 4, Day 7, Day 11, Day 14, Day 18, Day 35, Day 70, and Day 98) to assess tumor progression and suppression in mice treated with adoptive T-cell transfer

therapy. Mice were divided into two groups: Akt2-OE (red mouse ID, n = 11) and control (black mouse ID, n = 10) based on their IVIS bioluminescence values at Day -2 (data not shown), ensuring comparable initial tumor burden across groups and received $2x10^5$ engineered CTLs at day 0. All images are shown for each time point, illustrating tumor burden differences among groups.

The yellow-starred (*) mice indicate cases where illness led to death after adoptive T-cell transfer, and these were excluded from the statistical analysis presented in Figure 2F. The red-starred (*) mice signify those whose tumors exceeded the size limit specified by the animal protocol or experienced a ≥20% weight loss, marking the experimental endpoint. The IVIS bioluminescence data for the mice were summarized and analyzed in **Figure 2F** to evaluate therapeutic efficacy and tumor control.

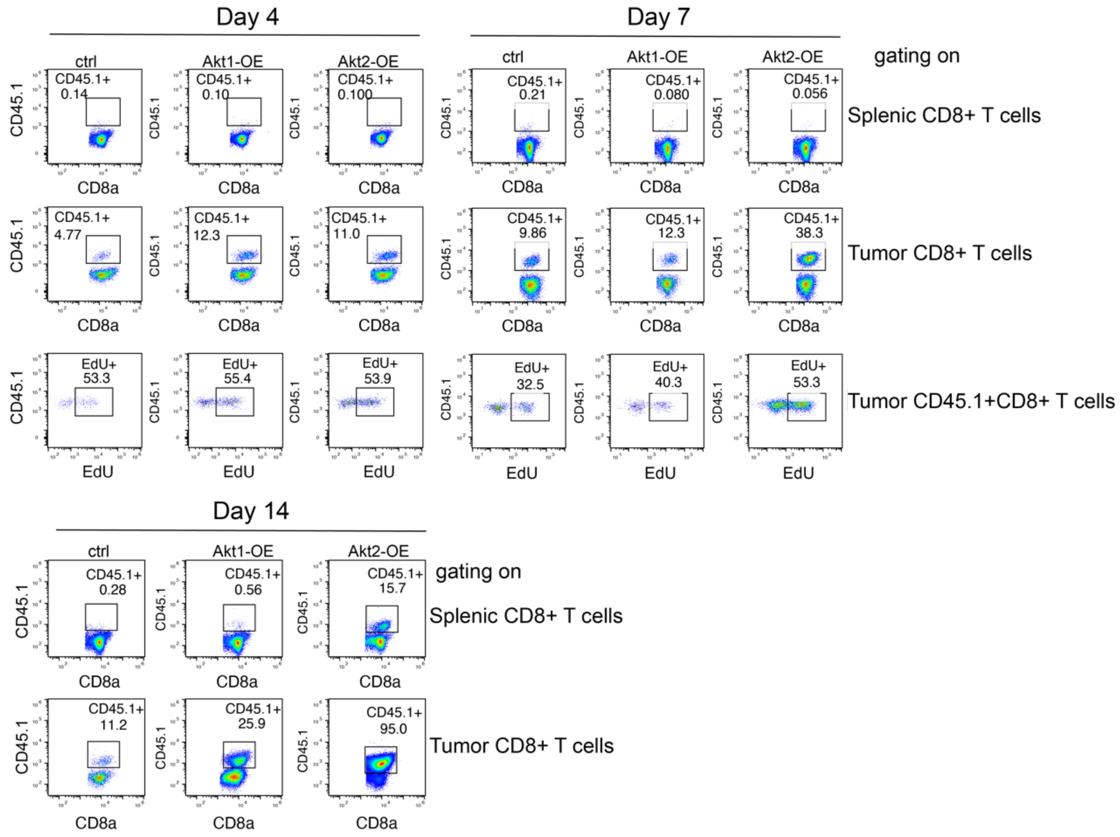

Figure S6. Time kinetics of adoptively transferred CD45.1⁺ CTLs and EdU incorporation across different *in vitro* engineered CTLs in tumor and spleen

Representative flow cytometry dot plots for **Figure 3A-3C**, showing the percentage of $CD45.1^+$ cells among total $CD8^+$ T cells and the proportion of EdU^+ (DNA synthesis) cells within $CD45.1^+$ CTLs in HCC-bearing mice after adoptive transfer of different *in vitro* engineered CTLs. Data are presented for the tumor and spleen at days 4, 7, and 11 post transfer. The results implied differences in the recruitment, expansion, and proliferation activity of distinct CTL in response to the tumor microenvironment. Percentages shown represent the gated populations in each dot plot.

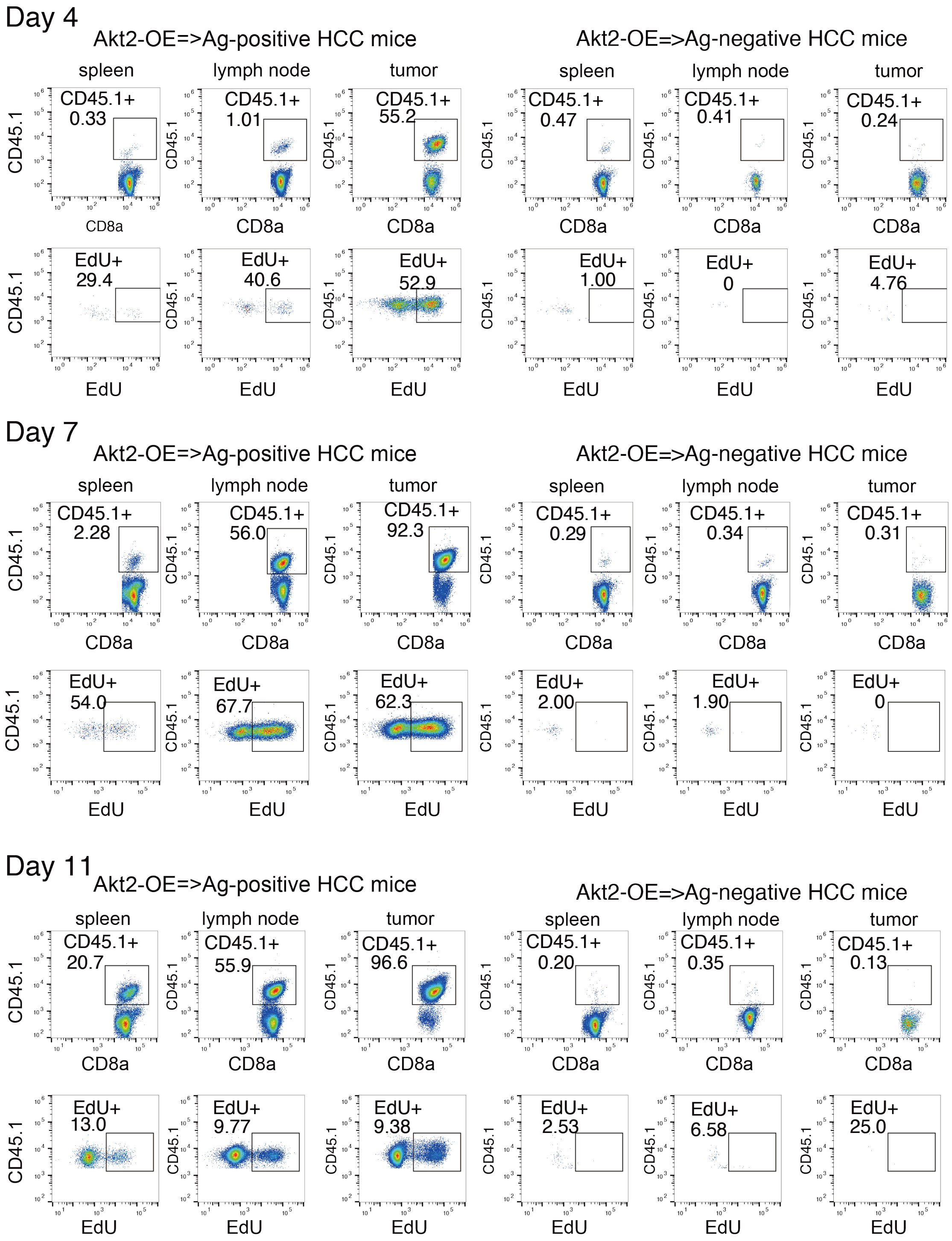

Figure S7. Antigen-dependent expansion and proliferation of Akt2-OE CTLs in HCC-bearing mice. Representative flow cytometry dot plots for **Figure 3D-3E**, showing the percentage of CD45.1^{+} cells among total CD8^{+} T cells and the proportion of EdU^{+} (DNA synthesis) cells within CD45.1^{+} CTLs in HCC-bearing mice with antigen-positive (Ag^{+}) or antigen-negative (Ag^{-}) tumors. Akt2-OE CTLs were adoptively transferred, and their recruitment, proliferation, and expansion were analyzed in the spleen, lymph nodes, and tumor tissues on days 4, 7, and 11 post-transfer. The data highlight that robust proliferation and expansion of Akt2-OE CTLs were observed only in Ag^{+} HCC mice, driven by antigen-dependent stimulation, while minimal activity was detected in Ag^{-} HCC mice. Percentages shown represent the gated populations in each dot plot.

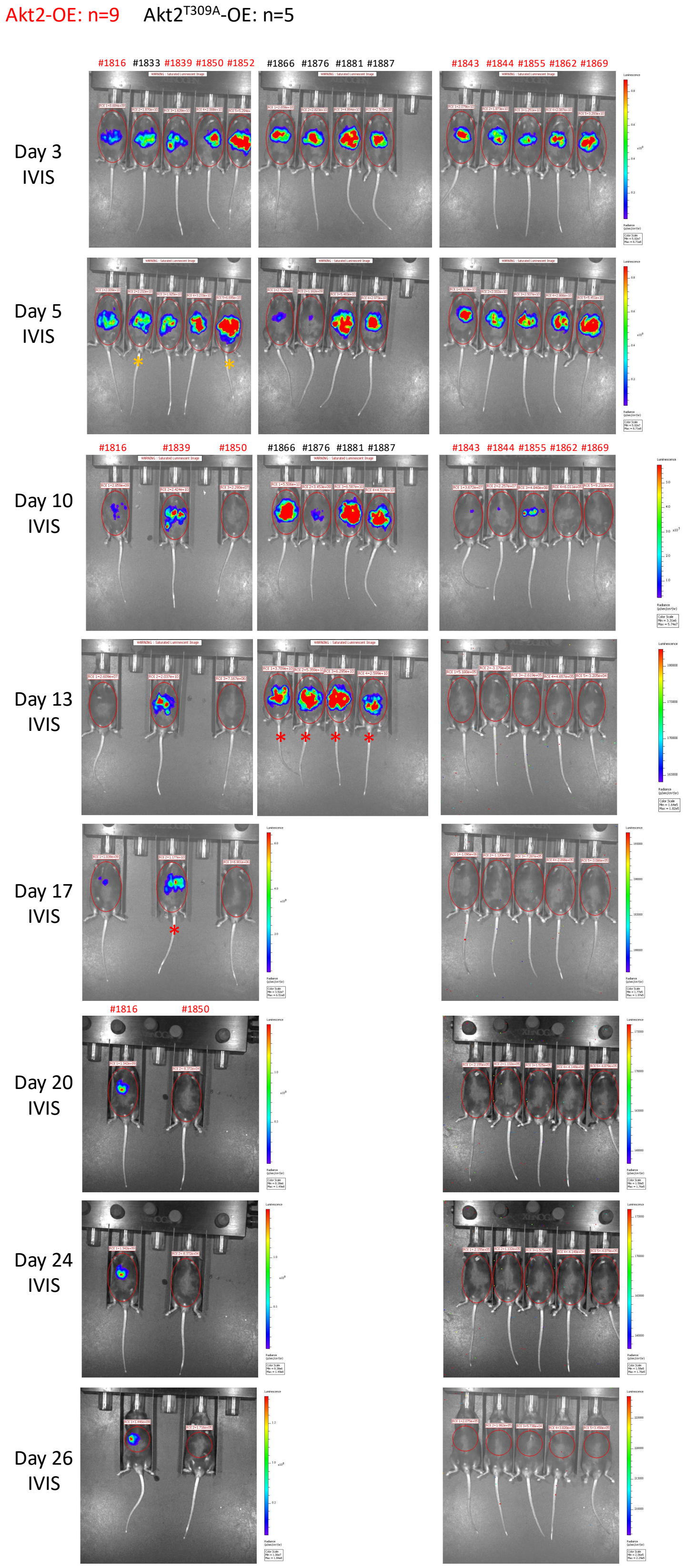

Figure S8. Longitudinal monitoring of tumor progression in mice receiving Akt2-OE or Akt2^{T309A}-OE CTLs.

IVIS imaging was performed at multiple time points (Day 3, Day 5, Day 10, Day 13, Day 17, Day 20, Day 24, and Day 26) to evaluate tumor progression in mice receiving adoptive transfer of 3.3 × 10^5 sorted Akt2-OE CTLs (n = 9) or Akt2^{T309A}-OE CTLs (n = 5). Mice were divided into two groups: Akt2-OE (red mouse

ID, n = 9) and Akt2^{T309A}-OE CTLs (black mouse ID, n = 5) based on their IVIS bioluminescence values at Day -2 (data not shown), ensuring comparable initial tumor burden across groups. All images are shown for each time point, illustrating tumor burden differences among groups.

The yellow-starred (*) mice indicate cases where illness led to death after adoptive T-cell transfer, and these were excluded from the statistical analysis presented in Figure 3F. The red-starred (*) mice signify those whose tumors exceeded the size limit specified by the animal protocol or experienced a ≥20% weight loss, marking the experimental endpoint. The IVIS bioluminescence data for all remaining mice were summarized and analyzed in **Figure 3F** to assess the therapeutic efficacy of Akt2-OE and Akt2^{T309A}-OE CTLs.

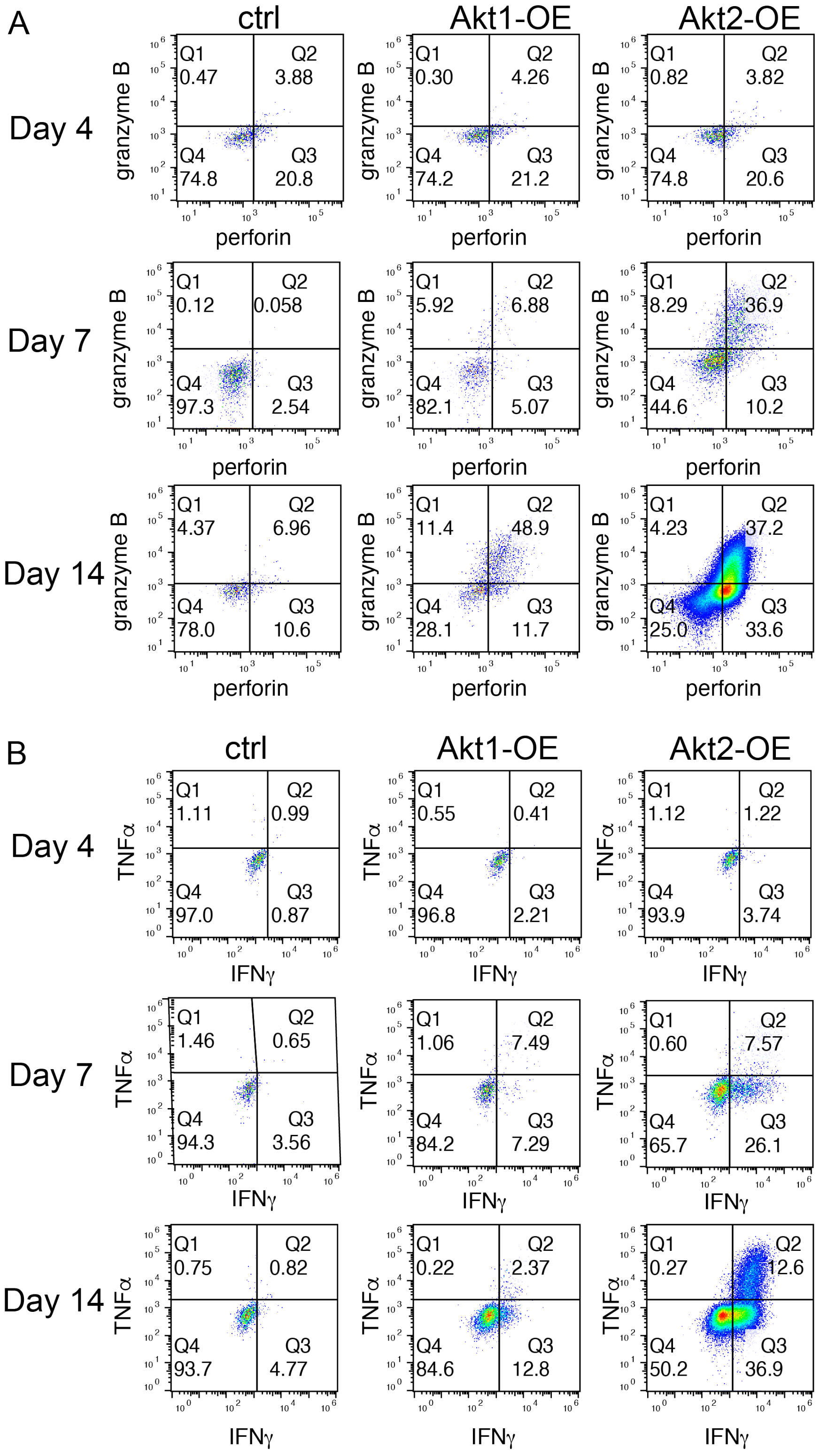

Figure S9. Effector function analysis of adoptively transferred CTLs in tumor tissues.

(A) Representative flow cytometry dot plots for Figure 4A-4C, showing the expression of perforin and granzyme B in $CD45.1^+$ CTLs isolated from tumor tissues of HCC-bearing mice at days 4, 7, and 14 post-adoptive transfer. **(B)** Representative flow cytometry dot plots for **Figure 4D-F**, illustrating the production of IFN-γ and TNF-α in $CD45.1^+$ CTLs after *ex vivo* stimulation with the cognate peptide $HBc_{93\text{-}100}$ at days 4, 7, and 14 post-adoptive transfer. These data demonstrate the effector function of adoptively transferred CTLs in the tumor microenvironment. Percentages shown represent the gated populations in each quadrant.

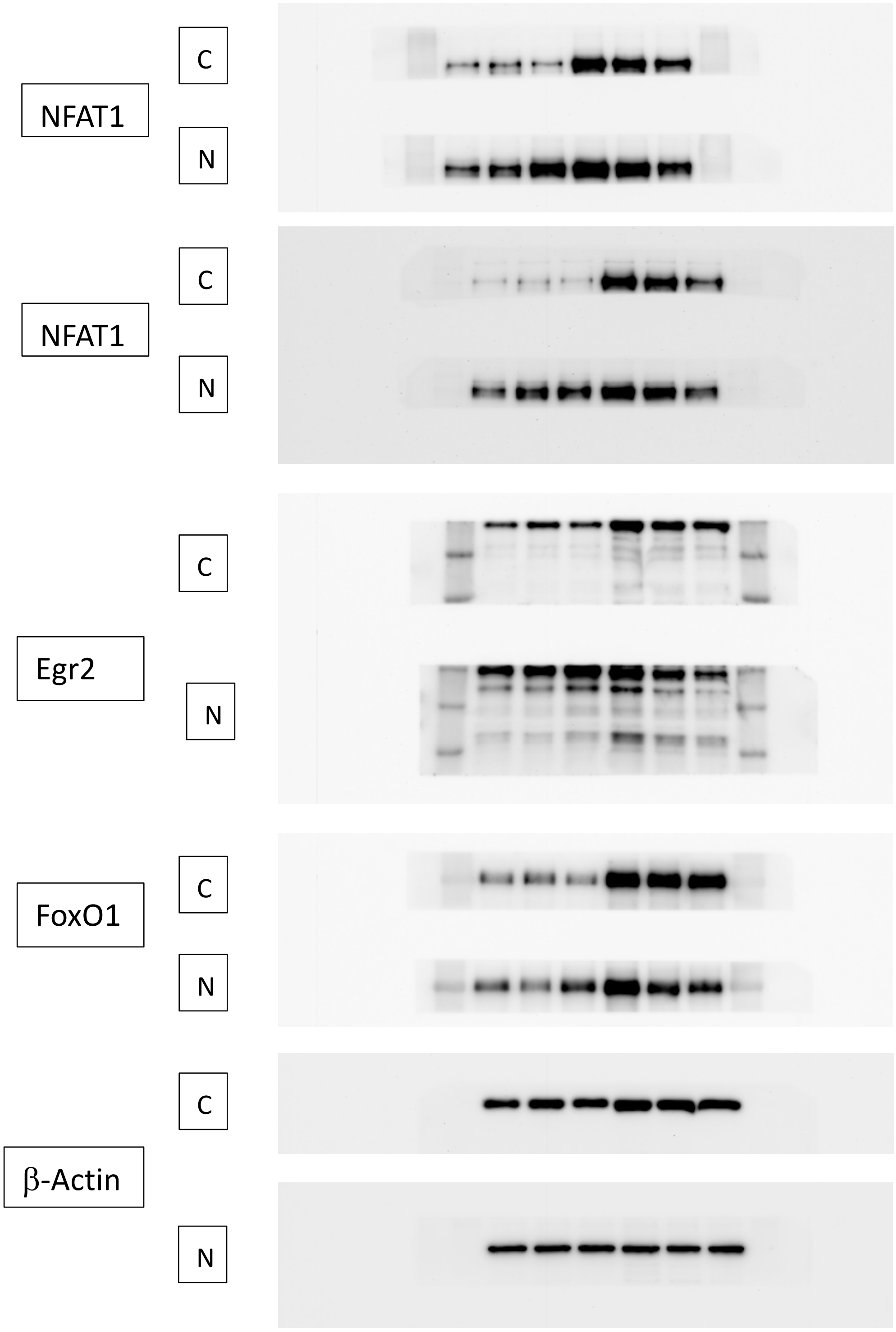

Figure S10. The uncut raw Western blot imaging of Figure 6A.

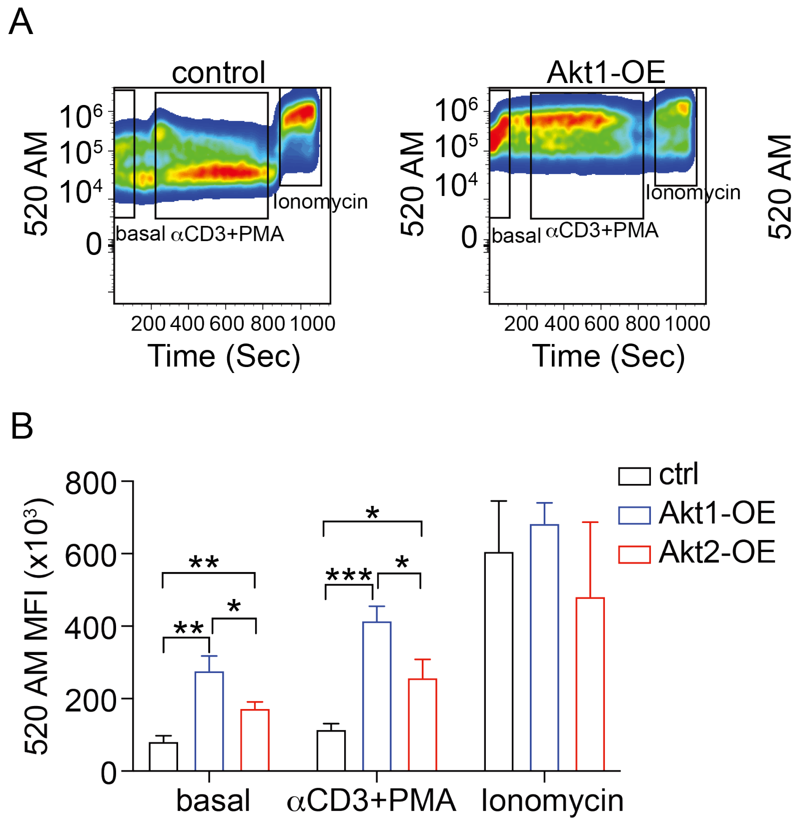

Figure S11. Akt2 signaling prevents calcium influx upon TCR triggering in Jurkat cells. (**A**) Kinetics of intracellular Ca^{2+} levels of control-, Akt1-OE, and Akt2-OE mouse CTLs upon aCD3 (10 mg/ml) +PMA (50 ng/ml) stimulation followed by ionomycin (0.5 ug/ml) treatment. (**B**) Histogram of Ca^{2+} levels of CTLs as in (A).

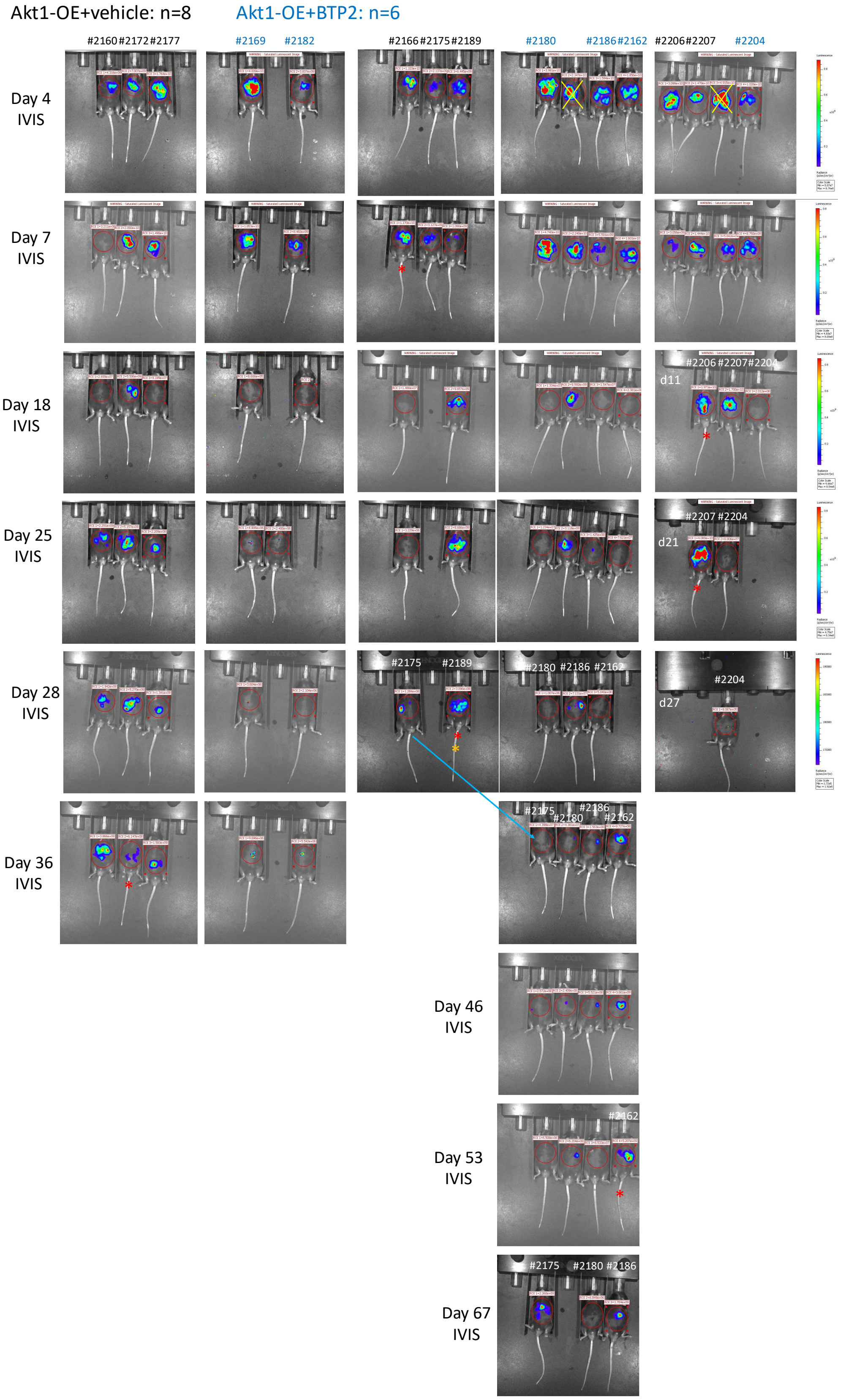

Figure S12. Longitudinal monitoring of tumor progression in mice receiving Akt1-OE CTLs pretreated with BTP2 (SOCC inhibitor).

IVIS images of all individual HCC-bearing mice included in the analysis, taken at various time points after adoptive transfer of 1×10^6 DMSO (vehicle)-treated Akt1-OE CTLs (black mouse ID, n=8) or BTP2-treated Akt1-OE CTLs (Blue mouse ID, n=6). Tumor progression was monitored on days 4, 7, 18, 25, 28, 36, 46, 53, and 67 post-treatment. For each mouse, tumor growth curves based on the detected IVIS signals are presented in **Figure 6I**, showing the kinetics of tumor progression for both treatment groups. The survival rate of mice in each group was analyzed and is summarized in **Figure 6J**, highlighting the improved survival associated with BTP2 pretreatment. The red-starred (*) mice signify those whose tumors exceeded the size limit specified by the animal protocol or experienced a ≥20% weight loss, marking the experimental endpoint. The red-starred mouse (*) exhibited discrepancies in IVIS signals, likely caused by tumor size interfering with luciferin absorption, resulting in inconsistencies between IVIS values and the liver tumor size observed after necropsy.

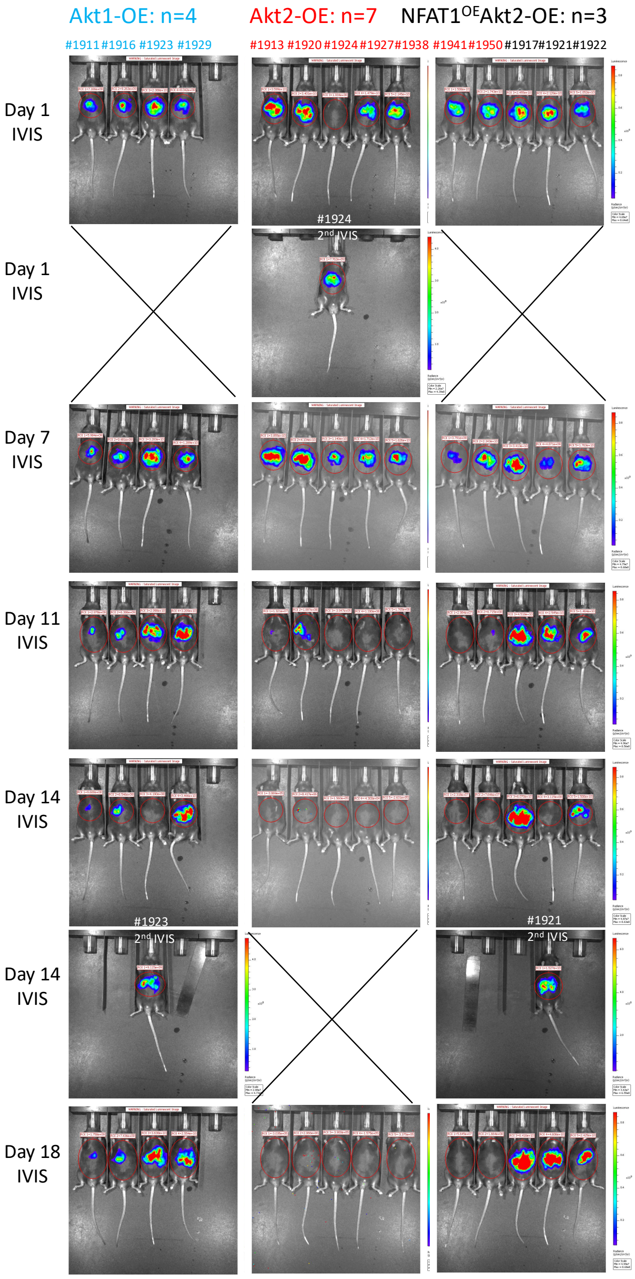

Figure S13. Longitudinal monitoring of tumor progression in mice receiving Akt1-OE, Akt2-OE or Akt2-OE co-expressing NFAT1 CTLs.

IVIS images of all individual HCC-bearing mice included in **Figure 7E**, taken at multiple time points after adoptive transfer of $4x10^5$ FACSorted Akt1-OE CTLs (blue mouse ID, n=4), Akt2-OE CTLs (red mouse ID, n=7), or NFAT1^{OE}Akt2-OE CTLs (black mouse ID, n=3). IVIS imaging was performed twice in some mice due to potential errors during the initial luciferin injection, which resulted in abnormally low signal values. These mice were re-injected with luciferin and re-imaged to ensure accurate data collection.

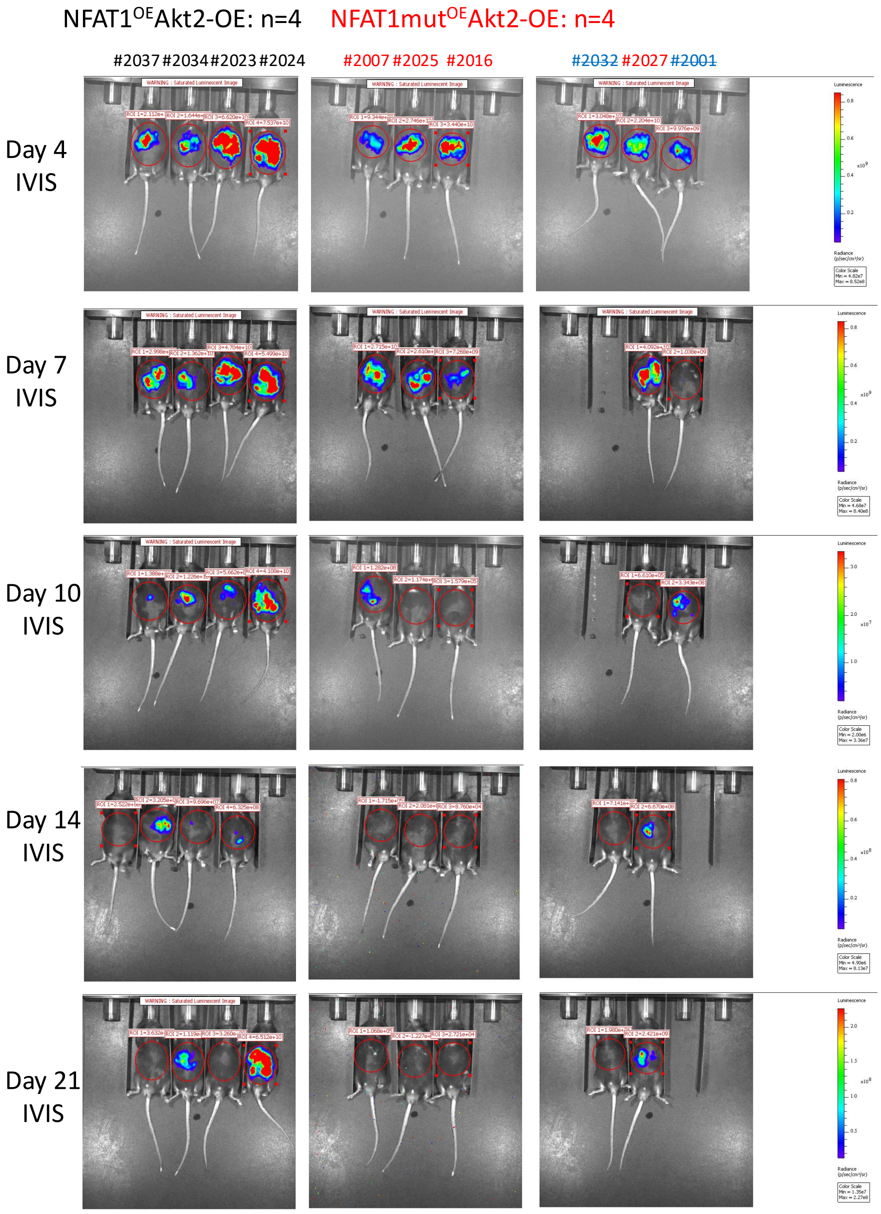

Figure S14. Longitudinal monitoring of tumor progression in mice receiving Akt2-OE co-expressing NFAT1 or NFAT1 mutant protein CTLs.

IVIS images of all individual HCC-bearing mice included in **Figure 7G**, taken at multiple time points after adoptive transfer of $4x10^5$ FACSorted NFAT1^{OE}Akt2-OE CTLs (black mouse ID, n=4) or NFATmut^{OE}Akt2-OE CTLs (red mouse ID, n=4).

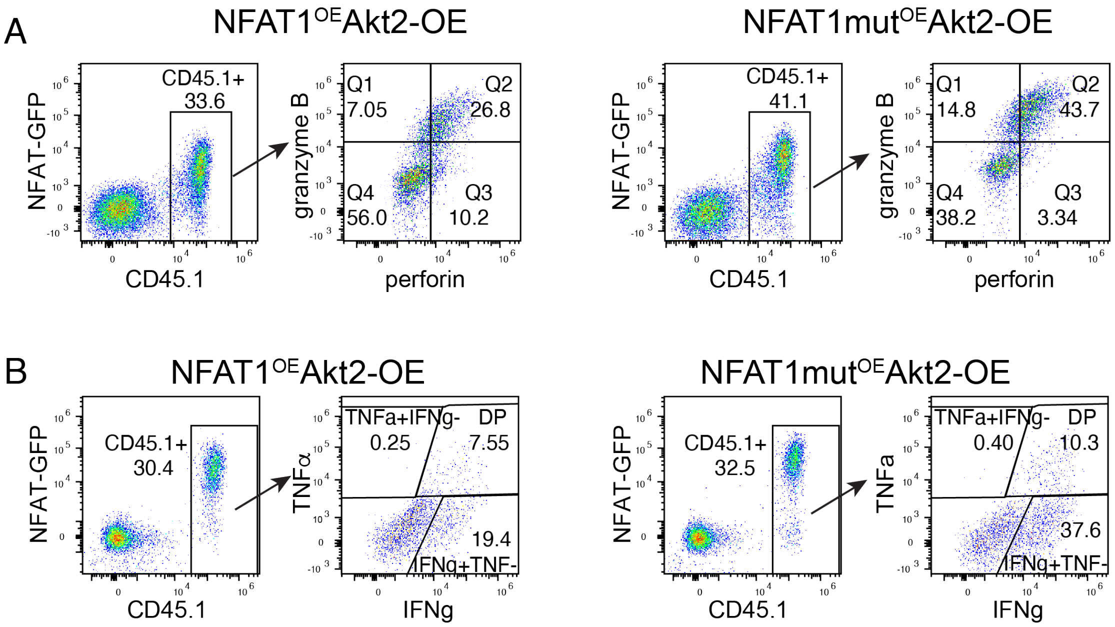

Figure S15. Functional profiling of NFAT1^{OE}Akt2-OE or NFAT1mut^{OE}Akt2-OE CTLs in tumor microenvironment.

Tumor-infiltrating lymphocytes (TILs) were analyzed 7 days after adoptive transfer of 4×10^5 FACSorted NFAT1^{OE}Akt2-OE or NFAT1mut^{OE}Akt2-OE CTLs into HCC tumor-bearing mice. Cells were first gated on CD8$^+$ T cells, followed by CD45.1$^+$ cells, as shown in the flow cytometry plots. The majority of CD45.1$^+$ cells were GFP-positive, confirming stable NFAT1 expression in the transferred cells. (**A**) Flow cytometry plots show surface marker expression followed by intracellular staining for perforin and granzyme B in CD45.1$^+$CD8$^+$ T cells. (**B**) Flow cytometry plots show intracellular staining for IFNγ and TNFα in CD45.1$^+$CD8$^+$ T cells. Quadrants represent populations positive for individual or dual expression of the respective markers (DP: double positive), and the numbers in each quadrant indicate the percentage of cells within the CD8$^+$CD45.1$^+$ gated population. These are representative plots; data from all mice in the experiment were summarized and analyzed in **Figure 7I** and **Figure 7J**.

Table S1. List of antibodies used in flow cytometry or immunohistochemistry

Antigen	Ab clone	Conjugation/isotype	Vendor
PD-1	J43	Fluorescein isothiocyanate (FITC)	Thermo Fisher Scientific,Waltham, USA
PD-1 (CD279)	J43	Brilliant Violet 605	BD Biosciences, CA, USA
LAG-3 (CD233)	C9B7W	Allophycocyanin (APC)	Biolegend, CA, USA
LAG-3	C9B7W	Brilliant Violet 421(BV421)	Biolegend
TIGIT	1G9	BV421	Biolegend
CD8α	53-6.7	Phycoerythrin (PE)	Biolegend
CD8α	53-6.7	PE-Cyanine7 (PE/Cy7)	Thermo Fisher Scientific
CD8α	53-6.7	PerCP-eFluor 710	Thermo Fisher Scientific
CD45.1	A20	PerCp-Cy5.5	Biolegend
CD45.1	A20	BV605	Biolegend
CD90.1	HIS51	PE	Thermo Fisher Scientific
CD90.1	OX-7	PerCp-Cy5.5	Biolegend
Perforin	S16009A	APC	Biolegend
Granzyme B	GB11	Pacific Blue	Biolegend
IFNγ	XMG1.2	FITC	Biolegend
TNFα	MP6-XT22	PE	Thermo Fisher Scientific
Fixable Viability dye	-	Zombie NIR™	Biolegend
CD45.1	A20	FITC/ Mouse IgG2a, κ	Biolegend
cleaved caspase 3	5A1E	Rabbit IgG	Cell Signaling Technology, Massachusetts, USA
F4/80	BM8	Rat IgG2a, κ	Biolegend
